# Primary efficacy of percutaneous microwave ablation of malignant liver tumors: comparison of stereotactic and conventional manual guidance

**DOI:** 10.1038/s41598-020-75925-6

**Published:** 2020-11-02

**Authors:** Jan Schaible, Lukas Lürken, Philipp Wiggermann, Niklas Verloh, Ingo Einspieler, Florian Zeman, Andreas G. Schreyer, Reto Bale, Christian Stroszczynski, Lukas Beyer

**Affiliations:** 1 Department of Radiology, University Medical Center Regensburg, 93053 Regensburg, Germany; 2 Department of Radiology and Nuclear Medicine, Hospital Braunschweig, Braunschweig, Germany; 3 Center for Clinical Studies, University Medical Center Regensburg, Regensburg, Germany; 4 Department of Radiology, Brandenburg Medical School, Brandenburg, Germany; 5 Department of Microinvasive Therapy (SIP), Medical University Innsbruck, Innsbruck, Austria

**Keywords:** Cancer, Cancer imaging

## Abstract

In this study, we compare the primary efficacy of computed tomography-navigated stereotactic guidance to that of manual guidance for percutaneous microwave ablation of liver malignancies. In total, 221 patients (140, 17, and 64 with hepatocellular carcinoma, cholangiocellular carcinoma, and liver metastases, respectively) with 423 treated liver lesions underwent microwave ablation (MWA). Manual guidance (M) and stereotactic guidance (S) were used for 136 and 287 lesions, respectively. The primary endpoint was the primary efficacy and the secondary endpoint was the radiation dose. A generalised estimating equation was applied to analyse the correlation between the primary efficacy (lesion basis) and the type of guidance, size and location of lesion. The primary efficacy rate was significantly higher in the S-group (84.3%) than in the M-group (75.0%, p = 0.03). Lesion size > 30 mm was negatively correlated with the efficacy rate (odds ratio 0.38; 95% confidence interval 0.20–0.74). Stereotactic guidance was associated with a significantly lower dose length product (p < 0.01). In this retrospective study, percutaneous microwave ablation under stereotactic guidance exhibited significantly greater primary efficacy than conventional manual guidance.

## Introduction

In recent years, percutaneous ablation of primary and secondary liver tumors has become an important alternative to surgical resection and is likely to occupy a more central role in the management of patients with hepatocellular carcinoma (HCC) and liver metastases from colorectal cancer (CRC)^1^.

One crucial factor during the procedure is the complete ablation of the malignant lesion with a sufficient safety margin. A study by Solbiati et al. showed that re-treatment of residual tumor tissue after ablation of colorectal metastasis significantly increased patient survival, thus emphasising the importance of complete tumor eradication^2^. Similarly, for HCC, an initial complete response to percutaneous ablation was associated with improved survival^3^.

Therefore, one predictive factor for patient survival is a high primary efficacy rate, which is defined as the percentage of tumor tissue that is successfully eradicated after the initial ablation procedure^3–5^. To achieve high primary efficacy, the ablation probes must be placed as precisely as possible in or around the tumor, which can be a major challenge when tumors are in difficult locations. A large-scale study with over 1000 ablations showed that the incorporation of computer-assisted targeting techniques limits operator dependency and optimises the results^6^. To achieve optimal probe placement, the improved visualisation of the lesion through software support, is a great advantage. Thus, both the anatomical situation and the expected ablation zone, in relation to the semi-automatically segmented tumor can be visualised in all spatial directions and also three-dimensionally (3D), which enables a correction or adjustment of the ablation settings, if necessary.

To our knowledge, no studies have compared primary efficacy rates between stereotactic ablation and conventional manual ablation. Therefore, the purpose of this study was to evaluate the primary efficacy of stereotactic ablation for malignant liver tumors and compare the findings with those for conventional manual ablation in a retrospective cohort.

## Results

### Patient and tumor characteristics

The baseline patient characteristics are summarised in Table 1. A total of 221 patients (179 male) were included in the study. The median age was 64 years (range 30–85). In total, 285 ablation sessions were performed with a median number of treatment sessions per patient of one (range 1–5) and 170 patients required one, 41 required two, and 10 required three or more sessions. A median of one (range 1–5) tumor was treated per ablation session.

**Table 1:** Baseline characteristics of patients with malignant liver tumors who underwent ablation using manual or stereotactic guidance.

	N = 221
**Age, years**	
Min.	30
Mean (sd)	64.51 (9.77)
Median (IQR)	64 (58.00, 72.00)
Max.	85
**Sex, n (%)**	
Male	179 (81)
Female	42 (19)
**Treated tumors per patient, n**	
Median (IQR)	1 (1.00, 2.00)
max	14
**Tumor entity, n (%)**	
HCC	140 (63)
CRC	42 (19)
CCC	17 (8)
Other	22 (10)

*Min.* minimum, *Max.* maximum, *IQR* interquartile range, *HCC* hepatocellular carcinoma, *CRC* colorectal cancer, *CCC* cholangiocellular carcinoma.

### Tumor characteristics

A total of 423 tumors spread across all liver segments were treated using microwave ablation (MWA) with either stereotactic (S) or manual guidance (M) (Table 2).The two most frequent tumor entities were HCC (n = 274) and CRC liver metastasis (n = 82), followed by cholangiocellular carcinoma CCC (n = 30).The median tumor size was 17 mm, with 61 tumors being 30 mm or larger.

**Table 2:** Characteristics of malignant liver tumors in patients who underwent ablation using manual or stereotactic guidance.

	Manual	Stereotactic	p
**Lesion size**			
< 3 cm	119 (87.5)	243 (84.7)	0.531
≥ 3 cm	17 (12.5)	44 (15.3)	
**Liver segment**			
I	2 (1.5)	6 (2.1)	0.200
II	15 (11.0)	38 (13.2)	
III	18 (13.2)	17 (5.9)	
IVa	18 (13.2)	27 (9.4)	
IVb	9 (6.6)	17 (5.9)	
V	16 (11.8)	37 (12.9)	
VI	21 (15.4)	38 (13.2)	
VII	15 (11.0)	42 (14.6)	
VIII	22 (16.2)	65 (22.6)	
**Subcapsular location**			
No	48 (35.3)	119 (41.5)	0.269
Yes	88 (64.7)	168 (58.5)	
**Subphrenic location**			
No	93 (68.4)	223 (77.7)	0.052
Yes	43 (31.6)	64 (22.3)	
**Proximity to vessel**			
No	106 (77.9)	199 (69.3)	0.084
Yes	30 (22.1)	88 (30.7)	
**Primary efficacy**			
Incomplete	34 (25.0)	45 (15.7)	0.030
Complete	102 (75.0)	242 (84.3)	

Subphrenic and subcapsular location are defined as a distance less than 10 mm to the diaphragm or liver capsule.

*IQR* interquartile range.

### Primary efficacy and prognostic factors

The primary efficacy rate using stereotactic guidance was significantly higher than that using manual guidance in both the univariable (p = 0.046) and multivariable model (p = 0.028). There was a negative correlation between increasing tumor size and efficacy rate (p ≤ 0.01).The results of generalized estimating equation GEE are shown in Table 3. Difficult to reach lesion locations or the proximity to large vessels were not predictive factors of primary efficacy.

**Table 3:** Generalised linear mixed model to analyse the influence of tumor and ablation characteristics on the primary efficacy rate.

Predictor	Univariable analysis			Multivariable analysis		
	OR	95% CI	p-value	OR	95% CI	p-value
**Guidance**						
Manual						
Stereotactic	1.79	1.01, 3.18	0.046	1.95	1.07, 3.55	0.028
**Lesion size**						
< 3 cm						
≥ 3 cm	0.44	0.23, 0.82	0.010	0.38	0.20, 0.74	0.004
**Tumor entity**						
HCC						
CRC	0.53	0.28, 1.00	0.048	0.47	0.24, 0.94	0.033
CCC	0.31	0.11, 0.85	0.023	0.31	0.11, 0.84	0.021
Other	1.16	0.40, 3.33	0.78	1.17	0.38, 3.59	0.79
Segments I, VII or VIII	1.36	0.77, 2.39	0.29	1.39	0.74, 2.61	0.31
Subphrenic location	1.00	0.59, 1.69	> 0.99	0.99	0.57, 1.71	0.97
Subcapsular location	1.20	0.72, 2.00	0.49	1.20	0.70, 2.06	0.51
Proximity to vessel	0.74	0.44, 1.27	0.28	0.81	0.48, 1.36	0.43

Subphrenic and subcapsular location are defined as a distance less than 10 mm to the diaphragm or liver capsule.

*OR* odds ratio, *95% CI* 95% confidence interval, *HCC* hepatocellular carcinoma, *CRC* colorectal cancer, *CCC* cholangiocellular carcinoma.

### Radiation dose

The mean dose length product (DLP) was 2633 µGy⋅ cm (range 679–9969 µGy cm). Stereotactic guidance was associated with a significantly lower DLP (Table 4).

**Table 4:** Linear mixed model to analyse the influence of ablation characteristics on the dose length product.

Predictor	Univariable analysis			Multivariable analysis		
	Beta	95% CI	p-value	Beta	95% CI	p-value
**Guidance**						
Manual						
Stereotactic	0.14	0.05, 0.24	0.003	− 542	− 825, − 260	< 0.001
Number of treated lesions in this session	0.00	− 0.05, 0.06	0.97	258	98, 417	0.002

### Procedural safety

In total, out of 423 MWA procedures, 248 (87.02%) were performed without any adverse events. Grade I and II complications (any deviation from the normal postinterventional course, e.g. fever or pain medication) occurred in 20 MWA procedures (7.02%). There was no significant difference in the frequency of complications (p = 0.75) between the groups. One patient died after MWA due to an accidental puncture of the pericardium with hemopericardium. The incidence of all documented complications is listed in Table 5.

**Table 5:** Frequency of adverse events categorised according to the Clavien-Dindo classification system.

	Stereotactic (N = 182)	Manual (N = 103)
**Complications, n (%)**		
None	157 (86)	91 (88)
I	16 (9)	4 (4)
II	3 (2)	4 (4)
III	5 (3)	1 (1)
IV	0 (0)	3 (3)
V	1 (1)	0 (0)

## Discussion

One predictive factor for patient survival is a high primary efficacy rate, which is defined as the percentage of successfully eradicated tumor tissue after the initial ablation procedure^3–5^.

The present study showed that the primary efficacy rate for stereotactic ablation was significantly higher than that for manual ablation (84.3% vs. 75.0%) in patients with malignant liver tumors. Lesion size > 30 mm and efficacy rate were negatively correlated (odds ratio (OR) 0.38; 95% confidence interval 0.20–0.74). Stereotactic guidance was associated with a significantly lower DLP (p < 0.01).

To achieve the most accurate probe position, navigation systems can be a helpful tool^7–13^. An accurate placement of the probe is a precondition for a high primary efficacy. A precise positioning of the probe, supported by a navigation software that improves visualization of the lesion, leads to complete eradication of the tumor and a sufficient safety margin. The improved display of the lesion and the expected ablation zone in all spatial directions and in 3D enables to optimize the ablation settings. Studies have also shown that the use of stereotactic navigation for liver ablation leads to a very high accuracy and primary efficacy, with a mean lateral error of 3.6 ± 2.5 mm at the needle tip, an angular error of 1.3° ± 1.2°, and a longitudinal error of − 7.4 ± 6.2 mm at the needle tip^14,15^. This method can also contribute to precise probe placement during other surgeries such as liver biopsy^16^ or ablation of osteoid osteomas^17^.

Our study demonstrated for the first time that the primary efficacy for malignant liver tumor ablation was significantly higher with stereotactic navigation (S-group) than with conventional fluoroscopic navigation (M-group). In fact, 84.3% of lesions in the S-group were completely ablated; this value was 75.0% in the M-group. However, there was a negative correlation between an increase in the tumor size and the primary efficacy rate (OR 0.38; 95% CI 0.20–0.74). In addition, the use of stereotactic navigation resulted in a significantly lower radiation dose (p < 0.01). This is consistent with the findings of previous studies on stereotactic ablation, which found that the total DLP was significantly lower in the S-group than in the M-group (2115 µGy⋅ cm (SD 276) vs. 3109 µGy⋅ cm (SD 1137), respectively; p < 0.01)^18^.

A potential limitation of our work is the long duration of ablations, factors affecting the ablation settings, and involvement of multiple specialists (three experienced radiologists L.P.B, L.L., and P.W.). Thus, it is possible that inter-user differences could have influenced our results or led to bias. Although the longer time span may have led to distortions, it has indeed ensured that a large number of patients could be included in this study. Furthermore, the ablations were performed by the same team during the entire time; hence, a high level of experience of the interventionalists can be assumed. There was also no significant change in the choice of the ablation systems. Over the entire period, the AcculisMicrowave Tissue Ablation (MTA) System (AngioDynamics, Latham, NY, USA), the Emprint™ Ablation System (Medtronic, Minneapolis, USA) and subsequently, the NeuWave Ablation System (Ethicon, Johnson & Johnson, Bridgewater New Jersey and Cincinnati, Ohio) were used. Until 2016 all ablations were performed manually. From 2016 onwards, the Cascination navigation system became available and was used whenever possible. The shorter period of navigation use, with a corresponding learning curve and better results, further supports the use of navigation for improved primary efficacy. The indications based on the BCLC guidelines have not changed over the years. In our opinion, despite the long time span, constant and comparable conditions can be assumed. Another limitation is the retrospective single-center study design. Nevertheless, we are convinced that the large sample size has provided valuable data with a consistently high level of quality.

To conclude, we demonstrated that the use of stereotactic navigation improved primary efficacy compared to conventional manual guidance. Considering the limitations mentioned above, for the first time, we have shown that SMWA might lead to a higher primary efficacy rate compared to non-navigated ablation. Future, prospective studies should focus on improving navigation systems so that the treatment of patients can be continuously optimized.

## Methods

### Study design and participant selection

The ethics committee of the University of Regensburg approved this retrospective study (approval no. 16-101-0137). All procedures performed in this study were in accordance with the ethical standards of our institution and with the 1964 Helsinki declaration and its later amendments. Patients gave written informed consent for use of their imaging and clinical data before the ablation procedure for research purposes. Written informed consent was obtained for publication of identifying information/images.

In all patients, the treatment plan was established by a multidisciplinary tumor board consisting of hepatologists, abdominal surgeons, oncologists, and interventional radiologists.

The decision to perform HCC ablation was based on Barcelona Clinic Liver Cancer guidelines (BCLC). Ablation was primarily performed on patients with very early and early HCC who were neither eligible for liver resection nor liver transplantation. Apart from a few individual cases discussed in the tumor conference, patients with single tumors smaller than 5 cm or up to three tumors smaller than 3 cm each, were treated.

Contraindications included vascular invasion, extrahepatic disease, or extrahepatic metastases that could not be resected. Colorectal liver metastases were treated when surgical resection was not possible.

The ablation registry of the University of Regensburg was searched for all patients who received percutaneous liver tumor ablation between 8/2011 and 04/2020. We included all patients treated with MWA with or without stereotactic navigation support.

The following parameters were assessed for both groups:

- Primary efficacy as determined by a lack of residual tumor tissue on the 6-week follow-up magnetic resonance (MRI) scans with a liver-specific contrast agent; the scans were evaluated by two experienced readers (L.B. [9 years of experience] and L.L. [8 years of experience]).
- Size of the tumor (long and short axes of the axial plane).
- Location of the tumor, determined through radiological assessments (computed tomography (CT)/MRI).
- DLP according to the dose report.
- Complications (during the procedures or postinterventional) as noted in the patient records and classified according to the Clavien-Dindo system.

All data generated or analysed during this study are included in this published article and its supplementary information files (Supplementary Table S1).

### Navigation system and ablation procedure

**Figure 1:**
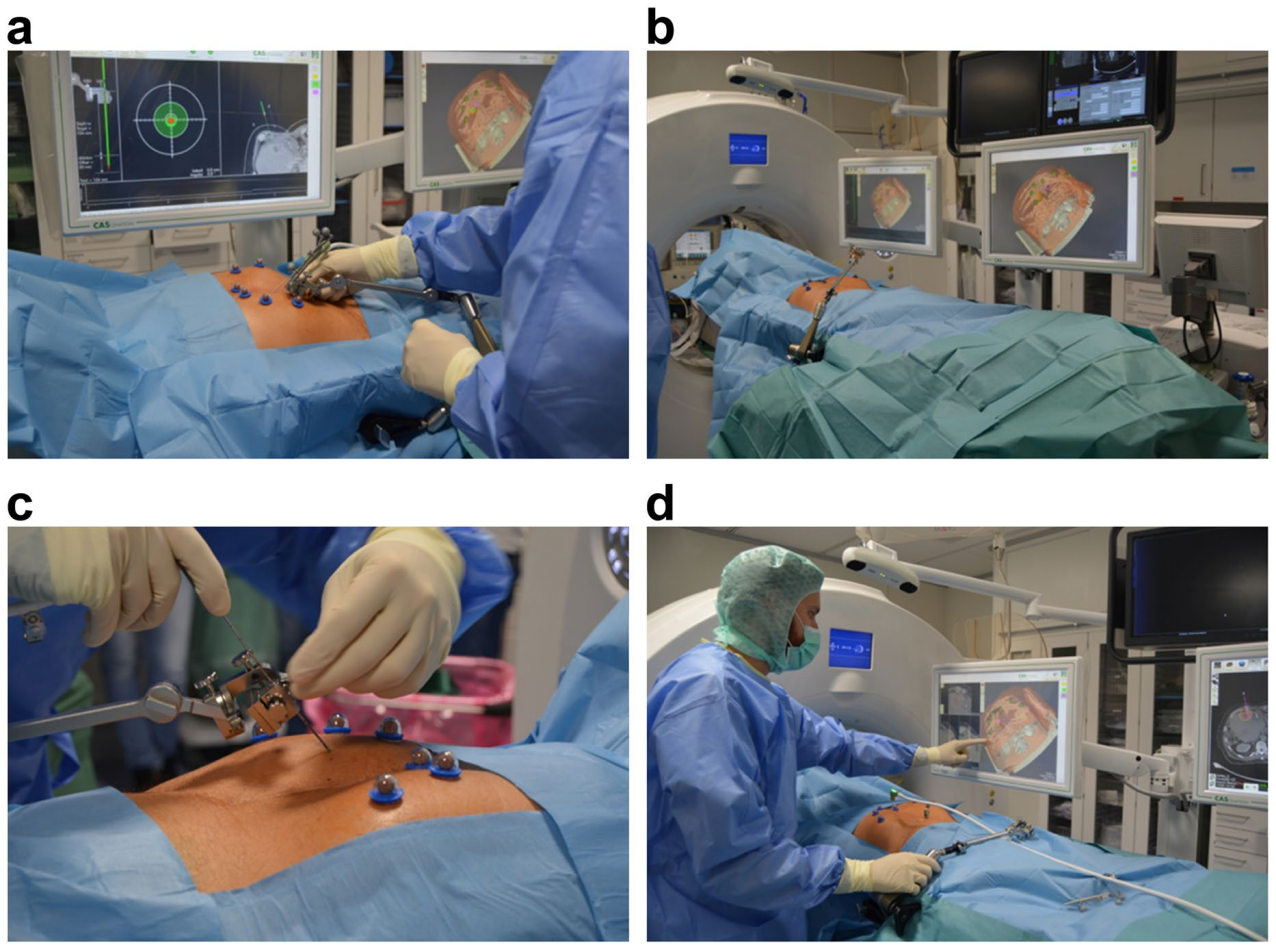
Setup and operation of the navigation system for ablation of malignant liver tumors. (**a**) A precise setting of the aiming device is crucial for optimal probe positioning. It is important that the positioning is always performed in apnoea, otherwise, major deviations must be expected. (**b**) Device setting and patient positioning: The computed tomography (CT)- and navigation-device-monitors are placed on the opposite side of the specialist. The tracking camera must be freely positioned to ensure optimal navigation. The aiming device is fixed on the side of the specialist. (**c**) The probe is fixed in the provided holder and the fine adjustment is made before puncturing the skin. It is important to place the optical markers outside the stitch area. (**d**) One of the two monitors should be covered in sterile material so that the specialist can check and readjust the optimal probe position directly.

All ablation procedures were performed under general anesthesia. Temporary apnoea was used during the planning CT scan and probe positioning to control respiratory movement in both groups (Fig. 1a).

In the S-group, sterile radiopaque reflective optical markers were attached to the patient after sterile scrubbing and draping. The tracking camera was fixed above the patient to track the markers and the aiming device (Fig. 1b).

An arterial and portal venous planning CT scan (Definition Edge or Somatom Sensation 16, Siemens Healthcare, Forchheim, Germany) was acquired under apnoea, and the data were sent to the navigation system (CAS-One IR, CAScination AG, Bern, Switzerland) adjacent to the CT gantry. If not automatically recognized, the spherical markers were manually identified. The registration was layer-based. However, if there were significant deviations due to different breathing positions, the registration was performed manually, by point-based method.

**Figure 2:**
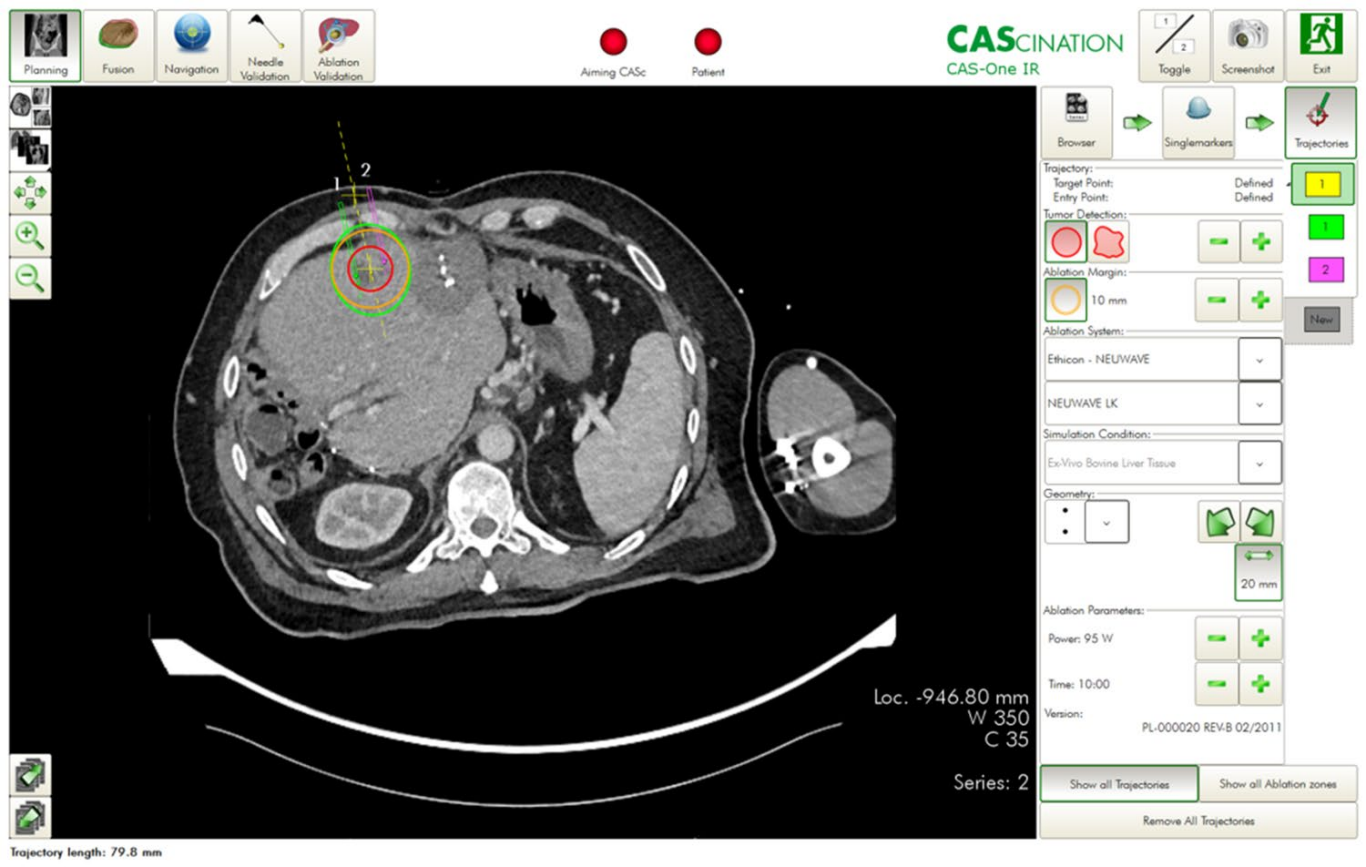
Planning of ablation and simulation of the ablation defect in patients with malignant liver tumors. The navigation software allows precise planning of the ablation. The tumor can be segmented in advance, and the resulting ablation defect can be determined with the appropriate safety distance.

**Figure 3:**
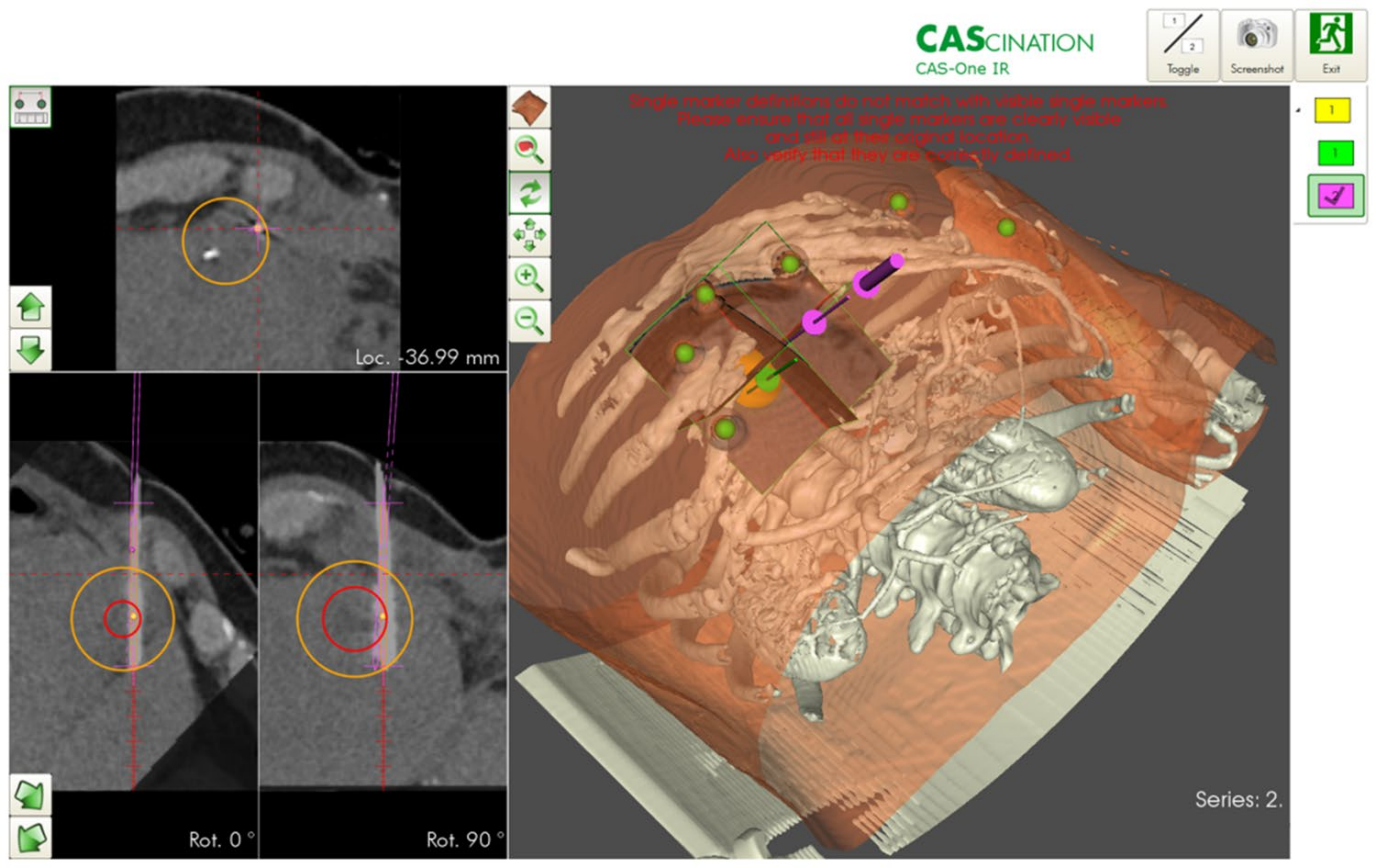
Verification of the correct probe position for ablation of malignant liver tumors. Before ablation, the needle position can be verified in all planes and corrected if necessary. The expected ablation defect can also be simulated. The software can vary the needle position, wattage, and ablation duration.

The desired ablation area and the target and entry points of the probe(s) were defined on multiplanar reconstructions of the 3D-CT datasets. The resulting trajectories were visualized on the 2D and 3D reconstructions accordingly (Fig. 2). The probes were then positioned using the CAScination aiming device, which allows locking of the probe axis relative to the patient (Fig. 1c). Before ablation, an unenhanced verification scan was performed and, if necessary, the probes were manually repositioned (Fig. 1d) and Fig. 3. Another native control scan was performed after completion of the ablation to exclude acute complications.

In the M-group, probe placement was achieved manually with repeated verifications using CT fluoroscopy after an initial 3-phase planning CT scan. CT fluoroscopy is an acquisition mode that allows continuous image updates using an in-room footswitch. During the ablation procedure, the probes were manually repositioned until the ablation area seemed to cover the tumor with a sufficient safety margin. If necessary, several overlapping ablations were performed.

For MWA, depending on tumor configuration and relationship to the surrounding tissue, either the AcculisMicrowave Tissue Ablation (MTA) System (AngioDynamics, Latham, NY, USA), the Emprint™ Ablation System (Medtronic, Minneapolis, USA), or the NeuWave Ablation System (Ethicon, Johnson & Johnson, Bridgewater New Jersey and Cincinnati, Ohio) was used. The navigation system was selected based on the specialists´ experience and the manufacturer’s. The Emprint system, for example, was used for round shaped tumors whereas the Accoulis was more likely used for large lesions with an ovoid configuration.

### Statistical analysis

Continuous data are presented as median, interquartile range, and data range, whereas categorical data are presented as absolute and relative frequencies. A GEE was applied to analyze the correlation between the primary efficacy (lesion basis) and the type of guidance (manual vs. stereotactic), size, and location of the lesion. As multiple lesions were ablated in the same patient, we included repeated measure analyses in our model. Adjusted odds ratios (ORs) and corresponding 95% confidence intervals (CIs) are reported as effect estimates. A two-sided p-value of < 0.05 was considered statistically significant. All analyses were performed using R 3.4.1 (R Foundation for Statistical Computing, Vienna, Austria).

## Supplementary information

Supplementary Information.

## Data Availability

All data generated or analysed during this study are included in this published article and its supplementary information files (Supplementary Table S1).
