# Supplementary material for: Primary efficacy of percutaneous microwave ablation of malignant liver tumors: comparison of stereotactic and conventional manual guidance
Source: Sci Rep. 2020 Nov 2;10:18835. doi: 10.1038/s41598-020-75925-6 (PMC7608621; doi:10.1038/s41598-020-75925-6)
Supplement: Supplementary file 1 — Supplementary Information. [file 41598_2020_75925_MOESM1_ESM.docx]

**Primary efficacy of percutaneous microwave ablation of malignant liver tumors:**

**comparison of stereotactic and conventional manual guidance**

*Dr. Jan Schaible, Dr. Lukas Lürken , Prof. Philipp Wiggermann , Dr. Niklas Verloh , Dr. Ingo Einspieler , Florian Zeman , Prof. Andreas G. Schreyer , Prof. Reto Bale , Prof. Christian Stroszczynski , PD Dr. Lukas Beyer*

**Supplementary Table S1**. Anonymized patient data

|  | complete | device | segment | long_axis | method | navigated | navsystem | subcapsular | subphrenic | vascular | pat_id | pat_entity | pat_male | pat_age | abl_id | abl_date | abl_ae | abl_aegrade | abl_lesion_count | abl_duration | abl_dlp | size | diff_segment |
| --- | --- | --- | --- | --- | --- | --- | --- | --- | --- | --- | --- | --- | --- | --- | --- | --- | --- | --- | --- | --- | --- | --- | --- |
| 2 | 0 | TATO | IVa | 62 | MWA | 1 | cascination | TRUE | FALSE | TRUE | rec0uXTUHAM3385VH | CCC | 0 | 61 | recV7ykA8FereI4kE | 18-06-19 | FALSE | None | 1 | 203 | 679 | large | FALSE |
| 3 | 0 | TATO | IVa | 33 | MWA | 1 | cascination | TRUE | FALSE | FALSE | rec0uXTUHAM3385VH | CCC | 0 | 61 | recFMURjgkxAMhS1R | 17-04-19 | FALSE | None | 1 | 75 | 1900 | large | FALSE |
| 4 | 1 | Emprint | VIII | 24 | MWA | 1 | cascination | TRUE | TRUE | FALSE | rec0VuOPIcegjbIiN | HCC | 0 | 71 | recK6nhTLBKu6GM4S | 29-05-19 | FALSE | None | 1 | 73 | 2025 | small | TRUE |
| 18 | 1 | Sort-A, Ilumark | VIII | 10 | MWA | 1 | cascination | TRUE | FALSE | TRUE | rec1egmRzvHc3Bbwc | HCC | 1 | 50 | reccM91HWDtXNLcZw | 29-11-18 | FALSE | None | 1 | 50 | 1246 | small | TRUE |
| 19 | 0 | Emprint | IVb | 21 | MWA | 1 | cascination | TRUE | FALSE | FALSE | rec1egmRzvHc3Bbwc | HCC | 1 | 50 | recaKRMGfLEamGMD8 | 30-04-19 | FALSE | None | 2 | 114 | 2592 | small | FALSE |
| 21 | 1 | Emprint | VIII | 20 | MWA | 1 | cascination | TRUE | FALSE | FALSE | rec1egmRzvHc3Bbwc | HCC | 1 | 50 | recaKRMGfLEamGMD8 | 30-04-19 | FALSE | None | 2 | 114 | 2592 | small | TRUE |
| 28 | 1 | Emprint | VI | 19 | MWA | 1 | cascination | TRUE | FALSE | FALSE | rec28si3RjlGi9T0t | HCC | 1 | 72 | recsVTRZmaXQS8q24 | 23-06-15 | TRUE | II | 1 | 72 | 2589 | small | FALSE |
| 35 | 1 | Emprint | III | 26 | MWA | 1 | cascination | TRUE | FALSE | TRUE | rec2PtcoA4EFUSGCS | Other | 1 | 65 | recQG6u19qyrQQGzM | 23-10-18 | FALSE | None | 1 | 82 | 1799 | small | FALSE |
| 37 | 1 | Emprint | V | 19 | MWA | 1 | cascination | TRUE | FALSE | FALSE | rec32sUQ5YQnEVjfJ | HCC | 1 | 75 | recCowdXSXRbzQQBt | 30-01-19 | FALSE | None | 1 | 84 | 2852 | small | FALSE |
| 38 | 1 | TATO | V | 7 | MWA | 1 | cascination | TRUE | FALSE | FALSE | rec32sUQ5YQnEVjfJ | HCC | 1 | 75 | recBozdIzTQ5n10y0 | 03-03-20 | FALSE | None | 1 | 60 | 1781 | small | FALSE |
| 39 | 1 | TATO | VII | 12 | MWA | 1 | cascination | FALSE | TRUE | FALSE | rec3c6jnlZCf3OeTV | Other | 1 | 60 | recj8dL94wHOXDTir | 01-08-19 | TRUE | III | 1 | 60 | 1781 | small | FALSE |
| 43 | 1 | SURGNOVA | VIII | 14 | MWA | 1 | cascination | FALSE | TRUE | FALSE | rec3jS7WqpCxbDRSz | HCC | 1 | 75 | recgb6eDclymm9DNz | 14-01-20 | FALSE | None | 2 | 135 | 2128 | small | TRUE |
| 45 | 1 | TATO | II | 13 | MWA | 1 | cascination | TRUE | TRUE | FALSE | rec46EIEzFLqzGwRM | HCC | 1 | 54 | recp1RDWXNzfQrFvt | 12-06-19 | FALSE | None | 2 | 106 | 2074 | small | FALSE |
| 46 | 1 | TATO | II | 22 | MWA | 1 | cascination | TRUE | FALSE | FALSE | rec46EIEzFLqzGwRM | HCC | 1 | 54 | recp1RDWXNzfQrFvt | 12-06-19 | FALSE | None | 2 | 106 | 2074 | small | FALSE |
| 54 | 1 | Emprint | IVa | 12 | MWA | 1 | cascination | FALSE | FALSE | FALSE | rec4JHIUGKyTKGKuS | HCC | 0 | 76 | recSAk0xf6sFGEKrM | 05-09-18 | FALSE | None | 1 | 72 | 1186 | small | FALSE |
| 55 | 0 | Emprint | VII | 19 | MWA | 1 | cascination | FALSE | FALSE | FALSE | rec4qiVxbUnVpTbzQ | HCC | 1 | 65 | recShVdaKghHlRbwK | 08-08-18 | FALSE | None | 1 | 75 | 1975 | small | TRUE |
| 56 | 1 | Emprint | VIII | 12 | MWA | 1 | cascination | FALSE | TRUE | FALSE | rec4qiVxbUnVpTbzQ | HCC | 1 | 65 | reckhyIwbBPp1Wccx | 28-03-18 | TRUE | I | 2 | 118 | 4720 | small | TRUE |
| 59 | 0 | Emprint | VII | 26 | MWA | 1 | cascination | FALSE | FALSE | FALSE | rec4qiVxbUnVpTbzQ | HCC | 1 | 65 | reckhyIwbBPp1Wccx | 28-03-18 | TRUE | I | 2 | 118 | 4720 | small | TRUE |
| 60 | 1 | Acculis | VIII | 42 | MWA | 1 | cascination | FALSE | FALSE | TRUE | rec4qiVxbUnVpTbzQ | HCC | 1 | 65 | recmuvrjfiqUdsn1g | 28-02-18 | FALSE | None | 1 | 133 | 3858 | large | TRUE |
| 61 | 1 | SURGNOVA | VI | 18 | MWA | 1 | cascination | TRUE | FALSE | FALSE | rec4rn2LxwK1Psqc4 | HCC | 1 | 73 | recWG9joSegDbs0u0 | 12-03-20 | FALSE | None | 1 | 60 | 1725 | small | FALSE |
| 62 | 1 | TATO | VIII | 33 | MWA | 1 | cascination | TRUE | FALSE | FALSE | rec4rn2LxwK1Psqc4 | HCC | 1 | 73 | recKS03ne0Anb7wW6 | 28-05-19 | FALSE | None | 1 | 125 | 1813 | large | TRUE |
| 65 | 1 | Emprint | VIII | 15 | MWA | 1 | cascination | FALSE | TRUE | FALSE | rec4Tg27JiBSf4s5e | HCC | 1 | 62 | reczGiPmP2hY26anE | 11-02-20 | FALSE | None | 2 | 108 | 3175 | small | TRUE |
| 66 | 1 | Emprint | VI | 29 | MWA | 1 | cascination | TRUE | TRUE | FALSE | rec4Tg27JiBSf4s5e | HCC | 1 | 62 | reczGiPmP2hY26anE | 11-02-20 | FALSE | None | 2 | 108 | 3175 | small | FALSE |
| 71 | 1 | Emprint | I | 15 | MWA | 1 | cascination | TRUE | FALSE | TRUE | rec5LpzJLAVXmOnKJ | HCC | 1 | 66 | recTC2RmkWPJiMnHD | 19-04-17 | FALSE | None | 1 | 61 | 2650 | small | TRUE |
| 78 | 1 | Emprint | IVb | 22 | MWA | 1 | cascination | TRUE | FALSE | FALSE | rec6B5RcuFBX5Hy4T | HCC | 1 | 63 | rec3s5oSAWXrGaXHD | 22-03-18 | FALSE | None | 1 | 65 | 3147 | small | FALSE |
| 91 | 1 | Acculis | V | 15 | MWA | 1 | cascination | FALSE | FALSE | FALSE | rec6Ufq4m1L5BvybE | HCC | 1 | 47 | recULSIHVnFRxty8y | 23-03-16 | FALSE | None | 1 | 67 | 3084 | small | FALSE |
| 94 | 0 | Emprint | VIII | 8 | MWA | 1 | cascination | FALSE | FALSE | TRUE | rec6utENz3J5eQE54 | CRC | 0 | 53 | recBGjeSEK63LYjKb | 01-04-20 | FALSE | None | 4 | 183 | 2272 | small | TRUE |
| 95 | 1 | Emprint | VII | 15 | MWA | 1 | cascination | FALSE | FALSE | FALSE | rec6utENz3J5eQE54 | CRC | 0 | 53 | recBGjeSEK63LYjKb | 01-04-20 | FALSE | None | 4 | 183 | 2272 | small | TRUE |
| 96 | 0 | Emprint | VII | 8 | MWA | 1 | cascination | FALSE | FALSE | FALSE | rec6utENz3J5eQE54 | CRC | 0 | 53 | recBGjeSEK63LYjKb | 01-04-20 | FALSE | None | 4 | 183 | 2272 | small | TRUE |
| 97 | 1 | Emprint | V | 16 | MWA | 1 | cascination | FALSE | TRUE | FALSE | rec6utENz3J5eQE54 | CRC | 0 | 53 | recBGjeSEK63LYjKb | 01-04-20 | FALSE | None | 4 | 183 | 2272 | small | FALSE |
| 103 | 1 | disposable Microwave Therapeutio | VI | 10 | MWA | 1 | cascination | FALSE | FALSE | TRUE | rec7MbQEwNpoUw0K0 | HCC | 1 | 52 | recKkbpM4EADEX9Ac | 05-04-19 | FALSE | None | 4 | 123 | 2672 | small | FALSE |
| 104 | 0 | Emprint | IVb | 19 | MWA | 1 | cascination | TRUE | FALSE | TRUE | rec7MbQEwNpoUw0K0 | HCC | 1 | 52 | recVDO8h59jaQu0HU | 15-05-18 | FALSE | None | 2 | 102 |  | small | FALSE |
| 105 | 1 | Emprint | IVa | 21 | MWA | 1 | cascination | FALSE | FALSE | TRUE | rec7MbQEwNpoUw0K0 | HCC | 1 | 52 | recVDO8h59jaQu0HU | 15-05-18 | FALSE | None | 2 | 102 |  | small | FALSE |
| 106 | 1 | disposable Microwave Therapeutio | V | 6 | MWA | 1 | cascination | TRUE | FALSE | TRUE | rec7MbQEwNpoUw0K0 | HCC | 1 | 52 | recKkbpM4EADEX9Ac | 05-04-19 | FALSE | None | 4 | 123 | 2672 | small | FALSE |
| 108 | 1 | disposable Microwave Therapeutio | VIII | 11 | MWA | 1 | cascination | FALSE | FALSE | FALSE | rec7MbQEwNpoUw0K0 | HCC | 1 | 52 | recKkbpM4EADEX9Ac | 05-04-19 | FALSE | None | 4 | 123 | 2672 | small | TRUE |
| 109 | 1 | disposable Microwave Therapeutio | I | 10 | MWA | 1 | cascination | FALSE | FALSE | TRUE | rec7MbQEwNpoUw0K0 | HCC | 1 | 52 | recKkbpM4EADEX9Ac | 05-04-19 | FALSE | None | 4 | 123 | 2672 | small | TRUE |
| 119 | 1 | Emprint | II | 15 | MWA | 1 | cascination | FALSE | FALSE | FALSE | rec8435ltXNVoCW1a | CRC | 1 | 69 | recWVGnY2jHHkAWY4 | 01-06-17 | FALSE | None | 1 |  |  | small | FALSE |
| 127 | 1 | Emprint | IVa | 25 | MWA | 1 | cascination | TRUE | FALSE | FALSE | rec8Pwru2Ri3y7c5e | HCC | 1 | 63 | recR3FyYe9HJOJtwG | 10-10-18 | FALSE | None | 1 | 69 | 2987 | small | FALSE |
| 137 | 1 | Acculis | II | 21 | MWA | 1 | cascination | TRUE | FALSE | FALSE | rec8YV1P1VAosNajR | HCC | 1 | 74 | reccaJJsjrQ1f2E5G | 09-02-16 | FALSE | None | 2 | 155 | 3542 | small | FALSE |
| 140 | 1 | Emprint | II | 14 | MWA | 1 | cascination | FALSE | FALSE | FALSE | rec9EOqUDugfqnJGV | HCC | 1 | 69 | recXvrIxcQa1mlJDP | 20-04-16 | FALSE | None | 3 | 100 | 3656 | small | FALSE |
| 173 | 1 | Emprint | II | 18 | MWA | 1 | cascination | TRUE | FALSE | TRUE | recaGRboGDhvQg4oe | HCC | 1 | 83 | recD21S8W9vPKKIc3 | 17-01-19 | FALSE | None | 4 | 104 | 2616 | small | FALSE |
| 174 | 1 | Emprint | VII | 11 | MWA | 1 | cascination | TRUE | FALSE | FALSE | recaGRboGDhvQg4oe | HCC | 1 | 83 | recD21S8W9vPKKIc3 | 17-01-19 | FALSE | None | 4 | 104 | 2616 | small | TRUE |
| 176 | 1 | Acculis | III | 39 | MWA | 1 | cascination | TRUE | FALSE | TRUE | recaGRboGDhvQg4oe | HCC | 1 | 83 | recYxut1fZbhMe4l8 | 30-01-18 | FALSE | None | 1 | 104 | 3678 | large | FALSE |
| 177 | 1 | Emprint | III | 23 | MWA | 1 | cascination | TRUE | TRUE | FALSE | recaGRboGDhvQg4oe | HCC | 1 | 83 | recD21S8W9vPKKIc3 | 17-01-19 | FALSE | None | 4 | 104 | 2616 | small | FALSE |
| 178 | 1 | TATO | V | 28 | MWA | 1 | cascination | TRUE | FALSE | FALSE | recAJnKmBSeSTjI1P | HCC | 1 | 75 | rec7OHtWrTmUSZyxp | 17-07-19 | TRUE | V | 3 | 222 | 2339 | small | FALSE |
| 179 | 1 | TATO | IVb | 41 | MWA | 1 | cascination | TRUE | FALSE | FALSE | recAJnKmBSeSTjI1P | HCC | 1 | 75 | rec7OHtWrTmUSZyxp | 17-07-19 | TRUE | V | 3 | 222 | 2339 | large | FALSE |
| 180 | 1 | TATO | V | 27 | MWA | 1 | cascination | TRUE | FALSE | TRUE | recAJnKmBSeSTjI1P | HCC | 1 | 75 | rec7OHtWrTmUSZyxp | 17-07-19 | TRUE | V | 3 | 222 | 2339 | small | FALSE |
| 191 | 1 | Emprint | VIII | 19 | MWA | 1 | cascination | FALSE | FALSE | TRUE | recAzkOXCzvwww7nP | HCC | 1 | 71 | recoqX6AbVpisu7kJ | 28-06-18 | TRUE | I | 1 | 115 | 1858 | small | TRUE |
| 198 | 1 | Acculis | VI | 21 | MWA | 1 | cascination | TRUE | FALSE | FALSE | recBhN9q9ZyAobQ8E | CRC | 1 | 65 | recYWEsbNtExS3Yhn | 12-02-20 | FALSE | None | 2 | 116 | 2321 | small | FALSE |
| 199 | 1 | Acculis | V | 14 | MWA | 1 | cascination | TRUE | TRUE | FALSE | recBhN9q9ZyAobQ8E | CRC | 1 | 65 | recYWEsbNtExS3Yhn | 12-02-20 | FALSE | None | 2 | 116 | 2321 | small | FALSE |
| 200 | 1 | TATO | VIII | 62 | MWA | 1 | cascination | TRUE | TRUE | FALSE | recbHx6gkFDSdQ1Mp | Other | 1 | 71 | recbMU42vQuIKZOB7 | 24-09-19 | FALSE | None | 1 | 136 | 2908 | large | TRUE |
| 203 | 1 | Emprint | VII | 23 | MWA | 1 | cascination | FALSE | FALSE | FALSE | recBMn48U4emdQyPS | HCC | 1 | 59 | recpD0mLtq889OyMM | 09-02-17 | TRUE | III | 2 | 116 | 2321 | small | FALSE |
| 204 | 0 | Emprint | VII | 27 | MWA | 1 | cascination | FALSE | FALSE | FALSE | recBMn48U4emdQyPS | HCC | 1 | 59 | recpD0mLtq889OyMM | 09-02-17 | TRUE | III | 1 | 136 | 2908 | large | TRUE |
| 208 | 0 | TATO | VII | 32 | MWA | 1 | cascination | FALSE | FALSE | TRUE | recBopq59xgb9MwEr | CCC | 1 | 80 | recxVRqydRmWvITca | 06-08-19 | FALSE | None | 3 | 169 | 2202 | large | TRUE |
| 209 | 0 | TATO | VIII | 23 | MWA | 1 | cascination | TRUE | FALSE | TRUE | recBopq59xgb9MwEr | CCC | 1 | 80 | recxVRqydRmWvITca | 06-08-19 | FALSE | None | 3 | 169 | 2202 | small | TRUE |
| 210 | 0 | TATO | VI | 20 | MWA | 1 | cascination | FALSE | FALSE | TRUE | recBopq59xgb9MwEr | CCC | 1 | 80 | recxVRqydRmWvITca | 06-08-19 | FALSE | None | 3 | 169 | 2202 | small | FALSE |
| 229 | 1 | Emprint | I | 16 | MWA | 1 | cascination | FALSE | FALSE | FALSE | recchZgJV1RV5qdfx | HCC | 1 | 55 | recxhg2HYYHSXdHk4 | 21-03-19 | TRUE | II | 2 | 126 | 3146 | small | TRUE |
| 230 | 1 | TATO | VII | 5 | MWA | 1 | cascination | FALSE | FALSE | FALSE | recchZgJV1RV5qdfx | HCC | 1 | 55 | recrRfmetbyQmZwUW | 31-07-19 | FALSE | None | 4 | 149 | 2832 | small | TRUE |
| 231 | 1 | TATO | VIII | 10 | MWA | 1 | cascination | TRUE | FALSE | FALSE | recchZgJV1RV5qdfx | HCC | 1 | 55 | recrRfmetbyQmZwUW | 31-07-19 | FALSE | None | 4 | 149 | 2832 | small | TRUE |
| 232 | 1 | TATO | II | 10 | MWA | 1 | cascination | TRUE | FALSE | FALSE | recchZgJV1RV5qdfx | HCC | 1 | 55 | recrRfmetbyQmZwUW | 31-07-19 | FALSE | None | 4 | 149 | 2832 | small | FALSE |
| 233 | 1 | Emprint | IVa | 13 | MWA | 1 | cascination | FALSE | FALSE | FALSE | recchZgJV1RV5qdfx | HCC | 1 | 55 | recxhg2HYYHSXdHk4 | 21-03-19 | TRUE | II | 2 | 126 | 3146 | small | FALSE |
| 234 | 0 | Acculis | IVa | 48 | MWA | 1 | cascination | FALSE | FALSE | TRUE | recchZgJV1RV5qdfx | HCC | 1 | 55 | recqzqcL0zEIyq539 | 21-02-19 | FALSE | None | 1 | 113 | 2275 | large | FALSE |
| 235 | 1 | TATO | VI | 7 | MWA | 1 | cascination | TRUE | FALSE | FALSE | recchZgJV1RV5qdfx | HCC | 1 | 55 | recrRfmetbyQmZwUW | 31-07-19 | FALSE | None | 4 | 149 | 2832 | small | FALSE |
| 236 | 1 | Emprint | III | 21 | MWA | 1 | cascination | FALSE | FALSE | FALSE | recCicwk8EwWe2KtX | HCC | 1 | 69 | recq9POXH0qIa0KqR | 31-07-18 | FALSE | None | 1 | 111 | 2266 | small | FALSE |
| 241 | 0 | Acculis | V | 44 | MWA | 1 | cascination | FALSE | FALSE | TRUE | reccKlVg8QrHNMLjJ | HCC | 1 | 84 | recyb3Cq6G2nEjwaH | 26-07-16 | FALSE | None | 1 | 189 | 3249 | large | FALSE |
| 247 | 1 | TATO | II | 8 | MWA | 1 | cascination | TRUE | TRUE | TRUE | reccPWXHD7SB1fs8n | HCC | 1 | 61 | recRCrsVULFsuwRD3 | 26-09-19 | FALSE | None | 1 | 54 | 1828 | small | FALSE |
| 248 | 1 | Emprint | IVb | 18 | MWA | 1 | cascination | TRUE | FALSE | FALSE | reccPWXHD7SB1fs8n | HCC | 1 | 61 | rec4NzeO2R0EBgVej | 07-03-19 | FALSE | None | 3 | 169 | 4353 | small | FALSE |
| 249 | 1 | Emprint | VIII | 14 | MWA | 1 | cascination | TRUE | FALSE | FALSE | reccPWXHD7SB1fs8n | HCC | 1 | 61 | rec4NzeO2R0EBgVej | 07-03-19 | FALSE | None | 3 | 169 | 4353 | small | TRUE |
| 250 | 1 | Emprint | III | 8 | MWA | 1 | cascination | TRUE | TRUE | FALSE | reccPWXHD7SB1fs8n | HCC | 1 | 61 | rec4NzeO2R0EBgVej | 07-03-19 | FALSE | None | 3 | 169 | 4353 | small | FALSE |
| 252 | 1 | Emprint | II | 28 | MWA | 1 | cascination | TRUE | FALSE | TRUE | recCtroVW16phETmu | CRC | 1 | 68 | recqk4Gyvn0bdCTjo | 14-03-18 | FALSE | None | 1 | 101 | 3617 | small | FALSE |
| 254 | 1 | Emprint | II | 17 | MWA | 1 | cascination | TRUE | TRUE | TRUE | reccVE0BwiQXsPTNY | HCC | 1 | 65 | recR0ZiYoHCT6mf60 | 28-01-19 | FALSE | None | 2 | 130 | 2883 | small | FALSE |
| 255 | 1 | Emprint | III | 9 | MWA | 1 | cascination | FALSE | FALSE | TRUE | reccVE0BwiQXsPTNY | HCC | 1 | 65 | recR0ZiYoHCT6mf60 | 28-01-19 | FALSE | None | 2 | 130 | 2883 | small | FALSE |
| 256 | 1 | Emprint | II | 17 | MWA | 1 | cascination | TRUE | TRUE | FALSE | reccVE0BwiQXsPTNY | HCC | 1 | 65 | rec0Mhie5EKJoNTKS | 29-06-18 | FALSE | None | 1 | 134 | 3283 | small | FALSE |
| 257 | 1 | TATO | III | 39 | MWA | 1 | cascination | FALSE | FALSE | FALSE | reccVE0BwiQXsPTNY | HCC | 1 | 65 | rec2nd8we5LvIJejt | 05-11-19 | FALSE | None | 1 | 61 | 3310 | large | FALSE |
| 260 | 1 | Acculis | VII | 34 | MWA | 1 | cascination | FALSE | FALSE | FALSE | recCy013HRivP3LNr | HCC | 1 | 67 | recqpDjGgdchL1LKl | 09-08-18 | FALSE | None | 1 | 142 | 2104 | large | TRUE |
| 261 | 1 | TATO | VI | 22 | MWA | 1 | cascination | TRUE | FALSE | TRUE | recCzu2L5LKjkisx1 | HCC | 1 | 65 | recjZPMRBot6fF7vN | 05-09-19 | FALSE | None | 2 | 135 | 2056 | small | FALSE |
| 262 | 1 | TATO | V | 22 | MWA | 1 | cascination | TRUE | FALSE | TRUE | recCzu2L5LKjkisx1 | HCC | 1 | 65 | recjZPMRBot6fF7vN | 05-09-19 | FALSE | None | 2 | 135 | 2056 | small | FALSE |
| 280 | 1 | Emprint | VII | 18 | MWA | 1 | cascination | TRUE | FALSE | FALSE | recDncMgmd6LsJEpN | HCC | 1 | 62 | recnPN43j4VXNO5Ew | 06-12-18 | TRUE | I | 4 | 149 | 2832 | small | FALSE |
| 281 | 1 | Emprint | VIII | 32 | MWA | 1 | cascination | FALSE | TRUE | FALSE | recDncMgmd6LsJEpN | HCC | 1 | 62 | recnPN43j4VXNO5Ew | 06-12-18 | TRUE | I | 4 | 149 | 2832 | large | FALSE |
| 282 | 1 | Emprint | III | 20 | MWA | 1 | cascination | FALSE | FALSE | TRUE | recDncMgmd6LsJEpN | HCC | 1 | 62 | recnPN43j4VXNO5Ew | 06-12-18 | TRUE | I | 4 | 149 | 2832 | small | FALSE |
| 285 | 1 | TATO | VIII | 34 | MWA | 1 | cascination | FALSE | FALSE | FALSE | recdVXKfFQc0m5GMR | CRC | 1 | 72 | recDnj4sN3TvDruFp | 06-11-19 | FALSE | None | 3 | 162 | 1773 | large | TRUE |
| 286 | 0 | TATO | V | 28 | MWA | 1 | cascination | TRUE | FALSE | FALSE | recdVXKfFQc0m5GMR | CRC | 1 | 72 | recDnj4sN3TvDruFp | 06-11-19 | FALSE | None | 3 | 162 | 1773 | small | FALSE |
| 287 | 1 | TATO | VIII | 31 | MWA | 1 | cascination | TRUE | FALSE | FALSE | recdVXKfFQc0m5GMR | CRC | 1 | 72 | recDnj4sN3TvDruFp | 06-11-19 | FALSE | None | 3 | 162 | 1773 | large | TRUE |
| 288 | 1 | Acculis | II | 15 | MWA | 1 | cascination | TRUE | FALSE | FALSE | recdYMnv2s1PxUzSW | HCC | 1 | 69 | recGc9WS7JKAjWhAI | 11-05-16 | TRUE | I | 2 | 86 | 3237 | small | FALSE |
| 289 | 1 | Emprint | V | 21 | MWA | 1 | cascination | FALSE | FALSE | TRUE | recdYMnv2s1PxUzSW | HCC | 1 | 69 | rec1PpF8BOVBtSzPQ | 27-04-17 | FALSE | None | 1 | 72 | 2701 | small | FALSE |
| 290 | 1 | Acculis | IVb | 30 | MWA | 1 | cascination | TRUE | FALSE | FALSE | recdYMnv2s1PxUzSW | HCC | 1 | 69 | recGc9WS7JKAjWhAI | 11-05-16 | TRUE | I | 2 | 86 | 3237 | small | FALSE |
| 293 | 1 | Emprint | IVa | 18 | MWA | 1 | cascination | TRUE | TRUE | FALSE | recEbg0ADfDF4LCyT | HCC | 1 | 61 | recVpIhxn9xZNfGsS | 06-02-18 | FALSE | None | 3 | 167 | 4036 | small | FALSE |
| 295 | 1 | Emprint | IVb | 16 | MWA | 1 | cascination | TRUE | FALSE | FALSE | recEbg0ADfDF4LCyT | HCC | 1 | 61 | recVpIhxn9xZNfGsS | 06-02-18 | FALSE | None | 3 | 167 | 4036 | small | FALSE |
| 297 | 1 | Emprint | V | 20 | MWA | 1 | cascination | TRUE | FALSE | TRUE | recEbg0ADfDF4LCyT | HCC | 1 | 61 | recVpIhxn9xZNfGsS | 06-02-18 | FALSE | None | 3 | 167 | 4036 | small | FALSE |
| 298 | 1 | Acculis | VIII | 35 | MWA | 1 | cascination | FALSE | FALSE | FALSE | reced2fAt8ubkekQc | HCC | 1 | 53 | rec24Fxd2uoXgckN6 | 07-08-18 | FALSE | None | 2 | 126 | 3759 | large | TRUE |
| 299 | 1 | Emprint | VII | 13 | MWA | 1 | cascination | TRUE | TRUE | FALSE | reced2fAt8ubkekQc | HCC | 1 | 53 | recHWZNxCa5YASXtg | 25-10-18 | FALSE | None | 1 | 84 | 1819 | small | TRUE |
| 300 | 1 | Emprint | VII | 24 | MWA | 1 | cascination | TRUE | FALSE | TRUE | reced2fAt8ubkekQc | HCC | 1 | 53 | rec24Fxd2uoXgckN6 | 07-08-18 | FALSE | None | 2 | 126 | 3759 | small | TRUE |
| 302 | 1 | TATO | VI | 16 | MWA | 1 | cascination | FALSE | FALSE | FALSE | recEQfd5LLGi0ilvk | Other | 1 | 63 | recUqT5QFH3KVjMKe | 30-07-19 | TRUE | III | 4 | 220 | 2809 | small | FALSE |
| 303 | 0 | TATO | VII | 21 | MWA | 1 | cascination | FALSE | FALSE | FALSE | recEQfd5LLGi0ilvk | Other | 1 | 63 | recUqT5QFH3KVjMKe | 30-07-19 | TRUE | III | 4 | 220 | 2809 | small | TRUE |
| 305 | 0 | TATO | VII | 13 | MWA | 1 | cascination | FALSE | FALSE | FALSE | recEQfd5LLGi0ilvk | Other | 1 | 63 | recUqT5QFH3KVjMKe | 30-07-19 | TRUE | III | 4 | 220 | 2809 | small | TRUE |
| 312 | 1 | Emprint | V | 13 | MWA | 1 | cascination | FALSE | FALSE | FALSE | recfg5kMGhMRDHi0r | HCC | 1 | 79 | rec37ICpfDGDzFiXl | 18-05-18 | FALSE | None | 2 | 104 | 2934 | small | FALSE |
| 314 | 1 | Emprint | V | 12 | MWA | 1 | cascination | TRUE | FALSE | TRUE | recfg5kMGhMRDHi0r | HCC | 1 | 79 | recaUD4ST8Kq6aM9Z | 17-10-18 | FALSE | None | 1 | 53 | 1746 | small | FALSE |
| 317 | 1 | Emprint | II | 10 | MWA | 1 | cascination | TRUE | FALSE | FALSE | recfg5kMGhMRDHi0r | HCC | 1 | 79 | rec37ICpfDGDzFiXl | 18-05-18 | FALSE | None | 2 | 104 | 2934 | small | FALSE |
| 329 | 1 | Emprint | VII | 17 | MWA | 1 | cascination | TRUE | TRUE | FALSE | recfu5PkZOquYaGRv | HCC | 1 | 59 | rec3lI7XyakgU8GOp | 23-11-17 | FALSE | None | 1 | 67 | 2667 | small | TRUE |
| 331 | 1 | TATO | VI | 15 | MWA | 1 | cascination | TRUE | FALSE | FALSE | recfwlwnZyWc18lDk | HCC | 1 | 81 | rec0qtX5eiQFDNQim | 12-06-19 | FALSE | None | 2 | 126 | 1627 | small | FALSE |
| 332 | 1 | Emprint | VI | 16 | MWA | 1 | cascination | FALSE | FALSE | FALSE | recfwlwnZyWc18lDk | HCC | 1 | 81 | recHOMie6AXNRqMCc | 14-08-19 | FALSE | None | 1 | 89 | 2175 | small | FALSE |
| 333 | 0 | TATO | VI | 36 | MWA | 1 | cascination | FALSE | FALSE | TRUE | recfwlwnZyWc18lDk | HCC | 1 | 81 | rec0qtX5eiQFDNQim | 12-06-19 | FALSE | None | 2 | 126 | 1627 | large | FALSE |
| 336 | 1 | Emprint | II | 9 | MWA | 1 | cascination | FALSE | FALSE | FALSE | recG20xZ19ZCluhKf | HCC | 1 | 57 | recAjyBs9D5qEZlqi | 14-06-18 | FALSE | None | 2 | 74 | 2460 | small | FALSE |
| 340 | 1 | Emprint | III | 12 | MWA | 1 | cascination | TRUE | FALSE | FALSE | recG20xZ19ZCluhKf | HCC | 1 | 57 | recpuwjPTXvSGYn7v | 13-04-16 | FALSE | None | 1 | 62 | 3805 | small | FALSE |
| 342 | 1 | Emprint | VII | 27 | MWA | 1 | cascination | TRUE | FALSE | TRUE | recG20xZ19ZCluhKf | HCC | 1 | 57 | recAjyBs9D5qEZlqi | 14-06-18 | FALSE | None | 2 | 74 | 2460 | small | TRUE |
| 355 | 1 | Acculis | II | 14 | MWA | 1 | cascination | FALSE | FALSE | FALSE | recGe2pjUrMDOAFCB | CRC | 1 | 50 | recu5FHWtNGpKyFzv | 10-08-16 | FALSE | None | 1 | 111 | 3211 | small | FALSE |
| 356 | 1 | TATO | VIII | 39 | MWA | 1 | cascination | TRUE | TRUE | FALSE | recgfn1MFnrb4Umvy | HCC | 1 | 68 | recBuS2rtfBj0awPy | 20-08-19 | FALSE | None | 1 | 142 | 1981 | large | TRUE |
| 360 | 1 | Emprint | II | 8 | MWA | 1 | cascination | TRUE | FALSE | FALSE | recgjyzVzcUg7lwz5 | Other | 0 | 73 | rec4abRy8yO23jwwZ | 11-05-16 | FALSE | None | 2 | 91 | 2993 | small | FALSE |
| 361 | 1 | Emprint | III | 6 | MWA | 1 | cascination | FALSE | FALSE | FALSE | recgjyzVzcUg7lwz5 | Other | 0 | 73 | rec4abRy8yO23jwwZ | 11-05-16 | FALSE | None | 2 | 91 | 2993 | small | FALSE |
| 369 | 1 | TATO | V | 33 | MWA | 1 | cascination | FALSE | FALSE | FALSE | recgMKoBiLaVl964r | HCC | 0 | 61 | reczYWvxUO2TEaN4R | 12-04-19 | FALSE | None | 1 | 148 | 4044 | large | FALSE |
| 378 | 1 | SURGNOVA | II | 13 | MWA | 1 | cascination | FALSE | FALSE | FALSE | recGqV83PdtMVt7de | HCC | 1 | 78 | recaz3Agh9C6Fh23M | 15-01-20 | FALSE | None | 2 | 208 | 2388 | small | FALSE |
| 379 | 1 | SURGNOVA | IVb | 53 | MWA | 1 | cascination | TRUE | TRUE | FALSE | recGqV83PdtMVt7de | HCC | 1 | 78 | recaz3Agh9C6Fh23M | 15-01-20 | FALSE | None | 2 | 208 | 2388 | large | FALSE |
| 380 | 1 | Emprint | IVa | 22 | MWA | 1 | cascination | TRUE | FALSE | TRUE | recGsxK47oePwaGxq | CRC | 0 | 59 | recmint7d6sXgRuyB | 22-10-19 | FALSE | None | 2 | 334 | 2056 | small | FALSE |
| 389 | 1 | Acculis | VIII | 10 | MWA | 1 | cascination | TRUE | TRUE | FALSE | recGXB8CEikwhdxHI | HCC | 1 | 54 | recJ9c2SOjQCpfcPn | 08-02-18 | FALSE | None | 2 | 167 | 4548 | small | TRUE |
| 395 | 1 | Emprint | VII | 18 | MWA | 1 | cascination | TRUE | FALSE | FALSE | recH6gBaMCM2aMzld | CRC | 1 | 57 | recrfZ3CZwhTFjDMB | 02-07-19 | FALSE | None | 1 | 81 | 2097 | small | TRUE |
| 401 | 1 | Emprint | IVa | 25 | MWA | 1 | cascination | FALSE | TRUE | FALSE | recHFOxjOsq5Ume1K | HCC | 1 | 62 | recbRK1moL9ej6nep | 23-01-19 | FALSE | None | 2 | 100 | 2078 | small | FALSE |
| 402 | 1 | Emprint | IVa | 14 | MWA | 1 | cascination | FALSE | FALSE | TRUE | recHFOxjOsq5Ume1K | HCC | 1 | 62 | recbRK1moL9ej6nep | 23-01-19 | FALSE | None | 2 | 100 | 2078 | small | FALSE |
| 403 | 1 | Acculis | V | 30 | MWA | 1 | cascination | TRUE | FALSE | FALSE | recHFOxjOsq5Ume1K | HCC | 1 | 62 | recvwrPWnOkRQkeYE | 08-11-18 | FALSE | None | 1 | 64 | 1765 | small | FALSE |
| 404 | 1 | Emprint | V | 13 | MWA | 1 | cascination | TRUE | TRUE | FALSE | recHFOxjOsq5Ume1K | HCC | 1 | 62 | recR7fQys5JMUWNy3 | 28-02-19 | FALSE | None | 1 | 62 | 2871 | small | FALSE |
| 410 | 1 | Emprint | VII | 16 | MWA | 1 | cascination | FALSE | FALSE | TRUE | recHKs3ESnwEo7E6Q | CRC | 0 | 72 | recfWK0H7j0M2VJvs | 12-11-19 | TRUE | I | 1 | 67 | 1096 | small | TRUE |
| 421 | 1 | Emprint | VII | 7 | MWA | 1 | cascination | FALSE | TRUE | FALSE | rechYlNLY7OWkPIBs | CCC | 1 | 69 | rec5PY5oxtIIgNIym | 30-05-18 | FALSE | None | 2 | 80 | 1517 | small | TRUE |
| 422 | 0 | Emprint | VIII | 10 | MWA | 1 | cascination | TRUE | FALSE | TRUE | rechYlNLY7OWkPIBs | CCC | 1 | 69 | rec5PY5oxtIIgNIym | 30-05-18 | FALSE | None | 2 | 80 | 1517 | small | TRUE |
| 426 | 1 | SURGNOVA | VIII | 10 | MWA | 1 | cascination | FALSE | FALSE | FALSE | recI1Nh2LZAjE82ad | CCC | 0 | 56 | recSWfiMcbMyZTHDK | 10-12-19 | FALSE | None | 2 | 102 | 1809 | small | TRUE |
| 427 | 1 | SURGNOVA | V | 18 | MWA | 1 | cascination | TRUE | TRUE | TRUE | recI1Nh2LZAjE82ad | CCC | 0 | 56 | recSWfiMcbMyZTHDK | 10-12-19 | FALSE | None | 2 | 102 | 1809 | small | FALSE |
| 428 | 1 | TATO | VII | 29 | MWA | 1 | cascination | TRUE | FALSE | FALSE | recICTZFkyj10fklk | HCC | 1 | 64 | rec5GqqBBSLOiD1Oo | 06-02-19 | FALSE | None | 1 | 63 | 1720 | small | TRUE |
| 429 | 1 | Acculis | V | 32 | MWA | 1 | cascination | FALSE | FALSE | TRUE | recICTZFkyj10fklk | HCC | 1 | 64 | recwtwhiTUdNWdkie | 15-11-18 | FALSE | None | 1 | 121 | 2666 | large | FALSE |
| 431 | 1 | Emprint | VI | 12 | MWA | 1 | cascination | TRUE | FALSE | FALSE | recikTuCJoQAFVaYX | HCC | 1 | 67 | rec6bwMfiKKmBTaVR | 21-08-18 | FALSE | None | 1 | 79 | 1908 | small | FALSE |
| 436 | 1 | TATO | VI | 33 | MWA | 1 | cascination | TRUE | FALSE | FALSE | reciUDwBoPQrSQEaj | Other | 1 | 57 | recC3hgpPegjbNnFK | 04-07-19 | FALSE | None | 2 | 217 | 3234 | large | FALSE |
| 437 | 1 | TATO | IVa | 38 | MWA | 1 | cascination | TRUE | FALSE | FALSE | reciUDwBoPQrSQEaj | Other | 1 | 57 | recC3hgpPegjbNnFK | 04-07-19 | FALSE | None | 2 | 217 | 3234 | large | FALSE |
| 441 | 1 | Acculis | VIII | 9 | MWA | 1 | cascination | TRUE | FALSE | FALSE | reciwpjaqLQXOlAx5 | HCC | 1 | 62 | rec6ouK6OsL9PGKFc | 16-03-16 | FALSE | None | 2 | 91 | 2686 | small | TRUE |
| 442 | 1 | Acculis | VIII | 9 | MWA | 1 | cascination | TRUE | TRUE | FALSE | reciwpjaqLQXOlAx5 | HCC | 1 | 62 | rec6ouK6OsL9PGKFc | 16-03-16 | FALSE | None | 2 | 91 | 2686 | small | TRUE |
| 444 | 1 | Acculis | VIII | 36 | MWA | 1 | cascination | FALSE | FALSE | TRUE | reciz4pZyINPhae6N | CCC | 1 | 67 | rec6qHHC74HBd8e3H | 29-03-18 | FALSE | None | 1 | 33 | 2141 | large | TRUE |
| 450 | 1 | Emprint | VIII | 10 | MWA | 1 | cascination | TRUE | FALSE | TRUE | recjGDA74dIRTKGqT | HCC | 1 | 59 | rec7xgSKDzCDPIGnN | 17-01-18 | FALSE | None | 2 | 271 | 5183 | small | TRUE |
| 451 | 1 | Emprint | VII | 18 | MWA | 1 | cascination | FALSE | FALSE | TRUE | recjI8UhObNAdRJYv | HCC | 1 | 58 | rec7zLcUnxHm9PJVp | 30-08-18 | FALSE | None | 1 | 46 | 2244 | small | TRUE |
| 452 | 1 | SURGNOVA | IVb | 8 | MWA | 1 | cascination | TRUE | TRUE | TRUE | recJIJjtTYZcjUiaH | HCC | 0 | 66 | recDWnw491A44N5X8 | 21-01-20 | FALSE | None | 1 | 73 | 1526 | small | FALSE |
| 461 | 1 | Emprint | VI | 8 | MWA | 1 | cascination | TRUE | TRUE | TRUE | recJPMTEdAB1qTks2 | HCC | 1 | 71 | recM7VgdwWENZBWfd | 02-10-18 | FALSE | None | 1 | 56 | 1587 | small | FALSE |
| 463 | 1 | Emprint | VI | 6 | MWA | 1 | cascination | TRUE | FALSE | TRUE | recJPMTEdAB1qTks2 | HCC | 1 | 71 | recLuXmK9Ckw8eUpZ | 23-01-18 | FALSE | None | 2 | 124 |  | small | FALSE |
| 464 | 1 | Acculis | VII | 46 | MWA | 1 | cascination | FALSE | FALSE | FALSE | recJPMTEdAB1qTks2 | HCC | 1 | 71 | recLuXmK9Ckw8eUpZ | 23-01-18 | FALSE | None | 2 | 124 |  | large | TRUE |
| 465 | 1 | Emprint | VIII | 14 | MWA | 1 | cascination | FALSE | TRUE | FALSE | recJPMTEdAB1qTks2 | HCC | 1 | 71 | recxGpbhMWvNmRkpW | 14-06-18 | FALSE | None | 3 | 101 | 2027 | small | TRUE |
| 468 | 1 | Emprint | I | 6 | MWA | 1 | cascination | TRUE | TRUE | TRUE | recJPMTEdAB1qTks2 | HCC | 1 | 71 | recxGpbhMWvNmRkpW | 14-06-18 | FALSE | None | 3 | 101 | 2027 | small | TRUE |
| 469 | 1 | Emprint | VIII | 11 | MWA | 1 | cascination | FALSE | FALSE | FALSE | recJPMTEdAB1qTks2 | HCC | 1 | 71 | recxGpbhMWvNmRkpW | 14-06-18 | FALSE | None | 3 | 101 | 2027 | small | TRUE |
| 476 | 1 | Emprint | II | 20 | MWA | 1 | cascination | FALSE | TRUE | TRUE | recJUxaodXtQeMlhB | HCC | 0 | 57 | rec7pVcw6XTLxnTj6 | 19-11-19 | TRUE | I | 2 | 155 | 2081 | small | FALSE |
| 477 | 1 | Emprint | IVa | 25 | MWA | 1 | cascination | FALSE | FALSE | FALSE | recJUxaodXtQeMlhB | HCC | 0 | 57 | recANRwk4yQDsXBFP | 18-09-19 | FALSE | None | 1 | 75 | 1782 | small | FALSE |
| 478 | 1 | Emprint | VI | 9 | MWA | 1 | cascination | TRUE | FALSE | FALSE | recJUxaodXtQeMlhB | HCC | 0 | 57 | rec7pVcw6XTLxnTj6 | 19-11-19 | TRUE | I | 2 | 155 | 2081 | small | FALSE |
| 482 | 1 | TATO | VIII | 12 | MWA | 1 | cascination | TRUE | FALSE | FALSE | reck1DL3L8iHtWCg0 | CRC | 1 | 61 | rec1nxUtkYDaN3cBI | 04-09-19 | FALSE | None | 5 | 139 | 1429 | small | TRUE |
| 483 | 1 | TATO | VII | 14 | MWA | 1 | cascination | FALSE | FALSE | TRUE | reck1DL3L8iHtWCg0 | CRC | 1 | 61 | rec1nxUtkYDaN3cBI | 04-09-19 | FALSE | None | 5 | 139 | 1429 | small | TRUE |
| 484 | 1 | TATO | VII | 13 | MWA | 1 | cascination | FALSE | TRUE | TRUE | reck1DL3L8iHtWCg0 | CRC | 1 | 61 | rec1nxUtkYDaN3cBI | 04-09-19 | FALSE | None | 5 | 139 | 1429 | small | TRUE |
| 485 | 1 | TATO | V | 10 | MWA | 1 | cascination | TRUE | FALSE | FALSE | reck1DL3L8iHtWCg0 | CRC | 1 | 61 | rec1nxUtkYDaN3cBI | 04-09-19 | FALSE | None | 5 | 139 | 1429 | small | FALSE |
| 486 | 0 | TATO | VI | 12 | MWA | 1 | cascination | FALSE | FALSE | TRUE | reck1DL3L8iHtWCg0 | CRC | 1 | 61 | rec1nxUtkYDaN3cBI | 04-09-19 | FALSE | None | 5 | 139 | 1429 | small | FALSE |
| 487 | 1 | TATO | VI | 10 | MWA | 1 | cascination | TRUE | FALSE | FALSE | reck3qJmIXU7tyqPm | Other | 1 | 77 | rec5jcptzLT5gUGPj | 20-11-19 | TRUE | I | 4 | 218 | 2588 | small | FALSE |
| 488 | 1 | TATO | VI | 13 | MWA | 1 | cascination | FALSE | FALSE | FALSE | reck3qJmIXU7tyqPm | Other | 1 | 77 | rec5jcptzLT5gUGPj | 20-11-19 | TRUE | I | 4 | 218 | 2588 | small | FALSE |
| 489 | 1 | TATO | V | 11 | MWA | 1 | cascination | TRUE | TRUE | FALSE | reck3qJmIXU7tyqPm | Other | 1 | 77 | rec5jcptzLT5gUGPj | 20-11-19 | TRUE | I | 4 | 218 | 2588 | small | FALSE |
| 490 | 1 | TATO | VIII | 8 | MWA | 1 | cascination | TRUE | FALSE | TRUE | reck3qJmIXU7tyqPm | Other | 1 | 77 | rec5jcptzLT5gUGPj | 20-11-19 | TRUE | I | 4 | 218 | 2588 | small | TRUE |
| 494 | 0 | Emprint | II | 19 | MWA | 1 | cascination | TRUE | FALSE | TRUE | recKiZbh6FVdGJZgS | CRC | 1 | 67 | reckfHeeMedttOe82 | 01-10-19 | FALSE | None | 3 | 77 | 977 | small | FALSE |
| 495 | 1 | Emprint | IVa | 28 | MWA | 1 | cascination | TRUE | FALSE | FALSE | recKiZbh6FVdGJZgS | CRC | 1 | 67 | reckfHeeMedttOe82 | 01-10-19 | FALSE | None | 3 | 77 | 977 | small | FALSE |
| 496 | 1 | Emprint | III | 16 | MWA | 1 | cascination | TRUE | TRUE | FALSE | recKiZbh6FVdGJZgS | CRC | 1 | 67 | reckfHeeMedttOe82 | 01-10-19 | FALSE | None | 3 | 77 | 977 | small | FALSE |
| 497 | 1 | Emprint | II | 13 | MWA | 1 | cascination | TRUE | FALSE | TRUE | recKiZbh6FVdGJZgS | CRC | 1 | 67 | recJqSGKgBZ4F8ahK | 05-12-19 | FALSE | None | 1 | 87 | 922 | small | FALSE |
| 498 | 1 | Emprint | V | 15 | MWA | 1 | cascination | TRUE | FALSE | FALSE | recKJvGaaLggczHvR | HCC | 1 | 73 | recyA8YNJ7a28xHsL | 23-03-16 | TRUE | II | 1 | 84 |  | small | FALSE |
| 503 | 1 | Emprint | VI | 12 | MWA | 1 | cascination | TRUE | FALSE | FALSE | recKKyA4MPL5T66NA | HCC | 1 | 73 | rec3CrH5px3C8tGq6 | 18-03-20 | FALSE | None | 1 | 54 | 1318 | small | FALSE |
| 513 | 1 | Emprint | V | 11 | MWA | 1 | cascination | TRUE | TRUE | FALSE | reckQSzfC8rdmK2pT | HCC | 0 | 58 | rec8HvRSbulZiI2mN | 27-03-18 | FALSE | None | 2 | 145 | 5211 | small | FALSE |
| 514 | 1 | Emprint | VIII | 21 | MWA | 1 | cascination | FALSE | FALSE | TRUE | reckQSzfC8rdmK2pT | HCC | 0 | 58 | rec8HvRSbulZiI2mN | 27-03-18 | FALSE | None | 2 | 145 | 5211 | small | TRUE |
| 515 | 1 | TATO | II | 28 | MWA | 1 | cascination | TRUE | FALSE | FALSE | reckQtVnoUvVsTYhd | HCC | 1 | 67 | recGu69TE4bJzIXen | 05-11-19 | FALSE | None | 1 | 116 | 1484 | small | FALSE |
| 522 | 1 | Emprint | IVa | 19 | MWA | 1 | cascination | TRUE | FALSE | TRUE | recKwJSvgGmwKVFYw | HCC | 1 | 56 | reckWy8uJBBSiOxOR | 13-12-18 | FALSE | None | 1 | 68 | 1687 | small | FALSE |
| 525 | 1 | Emprint | II | 17 | MWA | 1 | cascination | TRUE | FALSE | FALSE | reckXG9Qw3JGNn1lw | HCC | 0 | 85 | recUYIgPkeMtF3NIu | 06-02-20 | FALSE | None | 1 | 73 | 1221 | small | FALSE |
| 529 | 1 | Acculis | VII | 10 | MWA | 1 | cascination | TRUE | FALSE | FALSE | recL8p23G4FkLoj1m | CRC | 1 | 50 | reczZ2kGfqz6HmjYg | 04-12-18 | FALSE | None | 1 | 56 | 788 | small | TRUE |
| 531 | 1 | Acculis | V | 14 | MWA | 1 | cascination | FALSE | FALSE | TRUE | recL932TwU7MUhrW5 | HCC | 1 | 63 | recz0Gkw5g1yQfrTZ | 12-01-16 | FALSE | None | 2 | 142 | 3217 | small | FALSE |
| 533 | 1 | Acculis | IVa | 5 | MWA | 1 | cascination | TRUE | FALSE | FALSE | recL932TwU7MUhrW5 | HCC | 1 | 63 | recz0Gkw5g1yQfrTZ | 12-01-16 | FALSE | None | 2 | 142 | 3217 | small | FALSE |
| 534 | 1 | Acculis | II | 6 | MWA | 1 | cascination | FALSE | FALSE | FALSE | recL932TwU7MUhrW5 | HCC | 1 | 63 | recqEAgQSLelOpRKZ | 04-08-16 | FALSE | None | 2 | 49 |  | small | FALSE |
| 535 | 1 | Acculis | VIII | 8 | MWA | 1 | cascination | FALSE | FALSE | FALSE | recL932TwU7MUhrW5 | HCC | 1 | 63 | recqEAgQSLelOpRKZ | 04-08-16 | FALSE | None | 2 | 49 |  | small | TRUE |
| 537 | 1 | Emprint | I | 24 | MWA | 1 | cascination | TRUE | FALSE | TRUE | recLbKgIpCE6yddut | HCC | 1 | 54 | recz2nylYYySubdrn | 17-05-18 | FALSE | None | 1 | 130 | 4551 | small | TRUE |
| 541 | 1 | TATO | VII | 11 | MWA | 1 | cascination | TRUE | FALSE | FALSE | reclNqVmm2YkwZdFU | Other | 0 | 43 | recyh3Em93A7mHu6Y | 03-03-20 | FALSE | None | 1 | 96 | 1378 | small | TRUE |
| 550 | 1 | Emprint | IVa | 18 | MWA | 1 | cascination | FALSE | TRUE | FALSE | recM6aBceXXPATqrj | Other | 1 | 54 | recxZhFJBrk9mjWBW | 10-12-19 | FALSE | None | 1 | 96 | 3130 | small | FALSE |
| 551 | 1 | Emprint | VIII | 25 | MWA | 1 | cascination | TRUE | FALSE | FALSE | recM6aBceXXPATqrj | Other | 1 | 54 | recPuo5iTu87Rb7K7 | 09-10-19 | FALSE | None | 1 | 83 | 2078 | small | TRUE |
| 559 | 1 | Emprint | VII | 25 | MWA | 1 | cascination | FALSE | FALSE | TRUE | recmKLG0xr8KTQeaU | HCC | 1 | 65 | recZMWU1oVyN5BvGY | 28-08-19 | FALSE | None | 1 | 55 | 1520 | small | TRUE |
| 560 | 1 | Emprint | V | 10 | MWA | 1 | cascination | FALSE | FALSE | TRUE | recmKLG0xr8KTQeaU | HCC | 1 | 65 | recXk2Y8A95EzyHzy | 12-07-19 | FALSE | None | 2 | 138 | 2961 | small | FALSE |
| 561 | 1 | Emprint | VIII | 22 | MWA | 1 | cascination | TRUE | FALSE | FALSE | recmKLG0xr8KTQeaU | HCC | 1 | 65 | recXk2Y8A95EzyHzy | 12-07-19 | FALSE | None | 2 | 138 | 2961 | small | TRUE |
| 569 | 1 | Emprint | IVb | 25 | MWA | 1 | cascination | FALSE | TRUE | FALSE | recmw5OD8afRpasgV | CRC | 1 | 79 | recha5bGxvjtYMd8t | 17-04-19 | FALSE | None | 1 | 86 | 1325 | small | FALSE |
| 575 | 0 | Acculis | VII | 48 | MWA | 1 | cascination | TRUE | FALSE | TRUE | recNA7zBEjFG87EeK | HCC | 1 | 65 | rect6X4KfF2H20tXF | 27-04-16 | FALSE | None | 2 | 115 | 3665 | large | TRUE |
| 577 | 1 | Acculis | VIII | 33 | MWA | 1 | cascination | TRUE | TRUE | FALSE | recNA7zBEjFG87EeK | HCC | 1 | 65 | rect6X4KfF2H20tXF | 27-04-16 | FALSE | None | 2 | 115 | 3665 | large | TRUE |
| 580 | 1 | Emprint | IVa | 26 | MWA | 1 | cascination | TRUE | FALSE | FALSE | recni4vNXas0hgIci | HCC | 1 | 55 | recb9HNqwwmMdeI9c | 07-11-18 | FALSE | None | 3 | 206 | 2364 | small | FALSE |
| 581 | 1 | Emprint | IVa | 14 | MWA | 1 | cascination | FALSE | FALSE | FALSE | recni4vNXas0hgIci | HCC | 1 | 55 | recb9HNqwwmMdeI9c | 07-11-18 | FALSE | None | 3 | 206 | 2364 | small | FALSE |
| 582 | 1 | TATO | IVb | 17 | MWA | 1 | cascination | FALSE | FALSE | TRUE | recni4vNXas0hgIci | HCC | 1 | 55 | recivHuR8l6T6kNyo | 30-07-19 | FALSE | None | 1 | 53 | 1501 | small | FALSE |
| 583 | 1 | Emprint | III | 22 | MWA | 1 | cascination | FALSE | TRUE | TRUE | recni4vNXas0hgIci | HCC | 1 | 55 | recb9HNqwwmMdeI9c | 07-11-18 | FALSE | None | 3 | 206 | 2364 | small | FALSE |
| 584 | 1 | Emprint | VI | 16 | MWA | 1 | cascination | TRUE | FALSE | FALSE | recNImgNI3mzvPxHz | HCC | 1 | 56 | recVq0iejNlCZQeLY | 26-01-16 | FALSE | None | 1 | 69 | 2645 | small | FALSE |
| 589 | 1 | Acculis | VII | 85 | MWA | 1 | cascination | FALSE | FALSE | FALSE | recNjPejqJN16ODDg | CRC | 1 | 64 | recBaswWZ5HN2MDAa | 04-07-18 | FALSE | None | 2 | 96 | 1419 | large | TRUE |
| 590 | 1 | Emprint | VIII | 29 | MWA | 1 | cascination | TRUE | TRUE | FALSE | recNJTlLJqN9xL73O | HCC | 1 | 43 | recBAwDoiMHVtJ70I | 07-11-18 | FALSE | None | 2 | 124 | 1754 | small | TRUE |
| 591 | 1 | Acculis | VII | 40 | MWA | 1 | cascination | TRUE | FALSE | FALSE | recNJTlLJqN9xL73O | HCC | 1 | 43 | recBAwDoiMHVtJ70I | 07-11-18 | FALSE | None | 2 | 124 | 1754 | large | TRUE |
| 606 | 1 | Acculis | VI | 30 | MWA | 1 | cascination | TRUE | FALSE | FALSE | recntjECLawPB2IPI | CRC | 1 | 62 | recbkWWfkwqBx0IMC | 21-06-18 | FALSE | None | 1 | 73 | 1295 | small | FALSE |
| 614 | 1 | Emprint | V | 14 | MWA | 1 | cascination | FALSE | FALSE | FALSE | recnZvDJY2P3tCZB5 | HCC | 1 | 55 | recbQ8VmxoJPpAZyZ | 10-11-17 | FALSE | None | 3 | 112 | 3314 | small | FALSE |
| 616 | 0 | Emprint | VIII | 10 | MWA | 1 | cascination | TRUE | TRUE | FALSE | recnZvDJY2P3tCZB5 | HCC | 1 | 55 | recbQ8VmxoJPpAZyZ | 10-11-17 | FALSE | None | 3 | 112 | 3314 | small | TRUE |
| 617 | 1 | Emprint | IVb | 10 | MWA | 1 | cascination | FALSE | FALSE | FALSE | recnZvDJY2P3tCZB5 | HCC | 1 | 55 | recbQ8VmxoJPpAZyZ | 10-11-17 | FALSE | None | 3 | 112 | 3314 | small | FALSE |
| 620 | 1 | Emprint | IVa | 16 | MWA | 1 | cascination | TRUE | FALSE | TRUE | recoBkKMFf33r3G1W | CCC | 0 | 63 | reccsX2peBXPn1GYQ | 03-05-18 | TRUE | I | 3 | 110 | 3079 | small | FALSE |
| 621 | 1 | Emprint | V | 10 | MWA | 1 | cascination | TRUE | FALSE | FALSE | recoBkKMFf33r3G1W | CCC | 0 | 63 | reccsX2peBXPn1GYQ | 03-05-18 | TRUE | I | 3 | 110 | 3079 | small | FALSE |
| 622 | 1 | Emprint | VIII | 22 | MWA | 1 | cascination | TRUE | FALSE | FALSE | recoBkKMFf33r3G1W | CCC | 0 | 63 | reccsX2peBXPn1GYQ | 03-05-18 | TRUE | I | 3 | 110 | 3079 | small | TRUE |
| 629 | 1 | Acculis | VI | 33 | MWA | 1 | cascination | FALSE | FALSE | FALSE | recoiBHEOIDMsNIRm | Other | 1 | 75 | recc9eZhn4xyoLIOg | 05-07-18 | TRUE | I | 1 | 48 | 1144 | large | FALSE |
| 641 | 1 | Emprint | VI | 18 | MWA | 1 | cascination | TRUE | FALSE | TRUE | recoZrgIzf1TkfI6Y | CRC | 0 | 79 | reccQ4yl8BVFgdI3S | 13-11-18 | FALSE | None | 1 | 385 | 2787 | small | FALSE |
| 663 | 0 | Emprint | VI | 20 | MWA | 1 | cascination | TRUE | TRUE | FALSE | recPJJ91svAkPVS4t | CCC | 0 | 82 | recLkTCtbaoVu89M5 | 25-09-19 | FALSE | None | 1 | 53 | 1583 | small | FALSE |
| 664 | 1 | Emprint | VIII | 25 | MWA | 1 | cascination | TRUE | FALSE | FALSE | recPkAiVN9Z8c3OHe | CCC | 0 | 58 | recHjAA7VxavJua7S | 04-06-19 | FALSE | None | 2 | 81 | 3924 | small | TRUE |
| 666 | 1 | Emprint | VIII | 27 | MWA | 1 | cascination | TRUE | FALSE | TRUE | recPkAiVN9Z8c3OHe | CCC | 0 | 58 | recHjAA7VxavJua7S | 04-06-19 | FALSE | None | 2 | 81 | 3924 | small | TRUE |
| 667 | 0 | TATO | II | 40 | MWA | 1 | cascination | TRUE | FALSE | FALSE | recPkZZottO0ws2r9 | HCC | 1 | 75 | recVYQ1k9t826DYB0 | 23-10-19 | FALSE | None | 1 | 153 | 3079 | large | FALSE |
| 668 | 1 | SURGNOVA | II | 20 | MWA | 1 | cascination | FALSE | FALSE | FALSE | recPkZZottO0ws2r9 | HCC | 1 | 75 | recvRVkKtClUX7RLL | 08-01-20 | FALSE | None | 1 | 96 | 2610 | small | FALSE |
| 670 | 1 | Emprint | III | 10 | MWA | 1 | cascination | FALSE | FALSE | FALSE | recpMSuE5cJoaRoUE | HCC | 1 | 56 | recbAm6T20EBA9JwT | 28-04-16 | FALSE | None | 2 | 103 | 2636 | small | FALSE |
| 671 | 1 | Emprint | VIII | 8 | MWA | 1 | cascination | FALSE | FALSE | FALSE | recpMSuE5cJoaRoUE | HCC | 1 | 56 | recbAm6T20EBA9JwT | 28-04-16 | FALSE | None | 2 | 103 | 2636 | small | TRUE |
| 675 | 1 | Emprint | VIII | 19 | MWA | 1 | cascination | FALSE | TRUE | FALSE | recpMSuE5cJoaRoUE | HCC | 1 | 56 | recWgqNquOxdcSJPu | 30-03-16 | FALSE | None | 2 | 109 | 2636 | small | TRUE |
| 676 | 1 | Emprint | V | 11 | MWA | 1 | cascination | FALSE | FALSE | FALSE | recpMSuE5cJoaRoUE | HCC | 1 | 56 | recWgqNquOxdcSJPu | 30-03-16 | FALSE | None | 2 | 109 | 2636 | small | FALSE |
| 678 | 1 | TATO | VIII | 20 | MWA | 1 | cascination | FALSE | TRUE | TRUE | recPynymswkBAETAE | HCC | 0 | 68 | recD9iAonBUtZ6Yoh | 29-08-19 | FALSE | None | 1 | 80 | 1352 | small | TRUE |
| 679 | 0 | Emprint | V | 20 | MWA | 1 | cascination | TRUE | TRUE | FALSE | recPzLKFn468I0zF4 | CRC | 0 | 50 | recDqo2iWq0UEYzCY | 30-05-18 | FALSE | None | 3 | 112 | 4106 | small | FALSE |
| 680 | 1 | Emprint | III | 8 | MWA | 1 | cascination | TRUE | TRUE | FALSE | recPzLKFn468I0zF4 | CRC | 0 | 50 | recDqo2iWq0UEYzCY | 30-05-18 | FALSE | None | 3 | 112 | 4106 | small | FALSE |
| 681 | 1 | Emprint | V | 18 | MWA | 1 | cascination | FALSE | FALSE | FALSE | recPzLKFn468I0zF4 | CRC | 0 | 50 | reczI5NV1apmmjIcB | 01-08-18 | TRUE | I | 1 | 128 | 1670 | small | FALSE |
| 682 | 1 | Emprint | VIII | 7 | MWA | 1 | cascination | TRUE | FALSE | FALSE | recPzLKFn468I0zF4 | CRC | 0 | 50 | recDqo2iWq0UEYzCY | 30-05-18 | FALSE | None | 3 | 112 | 4106 | small | TRUE |
| 683 | 1 | TATO | II | 41 | MWA | 1 | cascination | FALSE | FALSE | FALSE | recq04mUGTLfUMYkr | CCC | 1 | 56 | recqXS8z2bQpjNgio | 03-04-19 | TRUE | III | 1 | 118 | 1550 | large | FALSE |
| 713 | 1 | Emprint | VI | 50 | MWA | 1 | cascination | TRUE | FALSE | TRUE | recRcmihMhGzISJ6s | HCC | 1 | 82 | reczvnJUxWPIUJpBG | 17-03-20 | FALSE | None | 1 | 98 | 1781 | large | FALSE |
| 734 | 1 | Emprint | VII | 24 | MWA | 1 | cascination | FALSE | FALSE | FALSE | recRVSSYwmbTCSj1N | HCC | 0 | 59 | recFMvaB5I5FyQjYH | 22-03-18 | FALSE | None | 1 | 82 | 3775 | small | TRUE |
| 744 | 1 | Emprint | VIII | 10 | MWA | 1 | cascination | TRUE | FALSE | TRUE | recSB6d6qh9o4Rtit | HCC | 1 | 66 | rec6QNLBPuquX1pcD | 10-05-19 | FALSE | None | 1 | 49 | 1177 | small | TRUE |
| 746 | 1 | Acculis | VII | 55 | MWA | 1 | cascination | FALSE | FALSE | FALSE | recSHLoc1XQZ8K1ma | CRC | 1 | 75 | recWoCtyLkY7fmvES | 06-11-18 | FALSE | None | 1 | 746 | 3096 | large | TRUE |
| 758 | 1 | Acculis | VII | 41 | MWA | 1 | cascination | TRUE | TRUE | FALSE | recsJ5jnRbVTRSQVI | HCC | 1 | 65 | recgAIB0qxPFNQQSC | 19-06-18 | FALSE | None | 1 | 70 | 2316 | large | TRUE |
| 759 | 1 | Emprint | VIII | 10 | MWA | 1 | cascination | FALSE | FALSE | FALSE | recSli5VVcgLBIHBG | CRC | 1 | 50 | recGcVnyuyaxxGHyA | 28-08-18 | TRUE | I | 2 | 109 | 3433 | small | TRUE |
| 760 | 1 | Emprint | VIII | 7 | MWA | 1 | cascination | TRUE | FALSE | FALSE | recSli5VVcgLBIHBG | CRC | 1 | 50 | recGcVnyuyaxxGHyA | 28-08-18 | TRUE | I | 2 | 109 | 3433 | small | TRUE |
| 761 | 1 | TATO | VIII | 17 | MWA | 1 | cascination | TRUE | FALSE | TRUE | recSQFWZIgJFacMXR | CRC | 1 | 45 | rec5LiD5OLWZISOVt | 11-09-19 | FALSE | None | 5 | 367 | 2727 | small | TRUE |
| 762 | 1 | TATO | VI | 20 | MWA | 1 | cascination | TRUE | FALSE | FALSE | recSQFWZIgJFacMXR | CRC | 1 | 45 | rec5LiD5OLWZISOVt | 11-09-19 | FALSE | None | 5 | 367 | 2727 | small | FALSE |
| 763 | 1 | TATO | VIII | 13 | MWA | 1 | cascination | FALSE | FALSE | FALSE | recSQFWZIgJFacMXR | CRC | 1 | 45 | rec5LiD5OLWZISOVt | 11-09-19 | FALSE | None | 5 | 367 | 2727 | small | TRUE |
| 764 | 0 | TATO | VII | 35 | MWA | 1 | cascination | TRUE | FALSE | TRUE | recSQFWZIgJFacMXR | CRC | 1 | 45 | rec5LiD5OLWZISOVt | 11-09-19 | FALSE | None | 5 | 367 | 2727 | large | TRUE |
| 765 | 0 | TATO | VIII | 10 | MWA | 1 | cascination | TRUE | FALSE | FALSE | recSQFWZIgJFacMXR | CRC | 1 | 45 | rec5LiD5OLWZISOVt | 11-09-19 | FALSE | None | 5 | 367 | 2727 | small | TRUE |
| 770 | 1 | TATO | VIII | 37 | MWA | 1 | cascination | TRUE | TRUE | TRUE | recSzW8PExXTgGNBv | CRC | 1 | 77 | recDEruZliLbNzUr8 | 03-09-19 | FALSE | None | 3 | 202 | 1841 | large | TRUE |
| 771 | 1 | TATO | VII | 46 | MWA | 1 | cascination | TRUE | FALSE | FALSE | recSzW8PExXTgGNBv | CRC | 1 | 77 | recDEruZliLbNzUr8 | 03-09-19 | FALSE | None | 3 | 202 | 1841 | large | TRUE |
| 772 | 1 | TATO | VII | 10 | MWA | 1 | cascination | FALSE | FALSE | TRUE | recSzW8PExXTgGNBv | CRC | 1 | 77 | recDEruZliLbNzUr8 | 03-09-19 | FALSE | None | 3 | 202 | 1841 | small | TRUE |
| 774 | 1 | Emprint | IVa | 26 | MWA | 1 | cascination | TRUE | FALSE | FALSE | recTeTAlBWL2uxHvw | Other | 1 | 72 | recH5wSYaiFOqvHsq | 24-10-18 | FALSE | None | 1 | 102 | 1468 | small | FALSE |
| 777 | 1 | Emprint | VII | 29 | MWA | 1 | cascination | TRUE | FALSE | FALSE | recTPF2RMPp1KbBjb | HCC | 1 | 82 | recHGikulbjNG9Bg5 | 29-08-18 | FALSE | None | 1 | 97 | 2051 | small | TRUE |
| 782 | 0 | TATO | IVa | 11 | MWA | 1 | cascination | TRUE | FALSE | TRUE | rectVFPW5gzb21g9I | HCC | 1 | 76 | recepg7Msx3QMxRZZ | 24-04-19 | FALSE | None | 4 | 168 | 2679 | small | FALSE |
| 783 | 0 | TATO | VI | 10 | MWA | 1 | cascination | FALSE | FALSE | FALSE | rectVFPW5gzb21g9I | HCC | 1 | 76 | recepg7Msx3QMxRZZ | 24-04-19 | FALSE | None | 4 | 168 | 2679 | small | FALSE |
| 784 | 1 | TATO | VIII | 10 | MWA | 1 | cascination | FALSE | FALSE | FALSE | rectVFPW5gzb21g9I | HCC | 1 | 76 | recepg7Msx3QMxRZZ | 24-04-19 | FALSE | None | 4 | 168 | 2679 | small | TRUE |
| 785 | 0 | TATO | I | 14 | MWA | 1 | cascination | TRUE | TRUE | FALSE | rectVFPW5gzb21g9I | HCC | 1 | 76 | recepg7Msx3QMxRZZ | 24-04-19 | FALSE | None | 4 | 168 | 2679 | small | TRUE |
| 789 | 0 | Emprint | VI | 28 | MWA | 1 | cascination | FALSE | FALSE | FALSE | recu3nYVn6dNMmafs | HCC | 1 | 53 | rec4whERkc7IjfdEE | 17-07-19 | FALSE | None | 1 | 79 | 1798 | small | FALSE |
| 790 | 0 | TATO | VI | 41 | MWA | 1 | cascination | FALSE | TRUE | FALSE | recu3nYVn6dNMmafs | HCC | 1 | 53 | recjo1IAIpfl61QLG | 06-11-19 | FALSE | None | 1 | 79 | 2640 | large | FALSE |
| 796 | 1 | TATO | VII | 35 | MWA | 1 | cascination | TRUE | FALSE | FALSE | recUBtVIgEPfbqDkf | HCC | 1 | 58 | recOynzWWJCI6sLeU | 03-12-19 | TRUE | I | 1 | 191 | 4895 | large | TRUE |
| 797 | 1 | TATO | V | 51 | MWA | 1 | cascination | TRUE | FALSE | TRUE | recUBtVIgEPfbqDkf | HCC | 1 | 58 | recUlVC0BI5GZagyQ | 18-12-19 | FALSE | None | 1 | 93 | 2853 | large | FALSE |
| 799 | 1 | TATO | V | 17 | MWA | 1 | cascination | FALSE | FALSE | FALSE | recuiOB5j6Xz5OgXZ | CRC | 1 | 64 | recCSuoVq22HZjaYH | 17-09-19 | FALSE | None | 1 | 63 | 1275 | small | FALSE |
| 802 | 1 | Emprint | VIII | 8 | MWA | 1 | cascination | TRUE | FALSE | FALSE | recULsp8CL03eFSK6 | CRC | 1 | 53 | recIC5HLb7UPaDSH0 | 23-05-18 | FALSE | None | 2 | 133 | 3764 | small | TRUE |
| 803 | 1 | Acculis | VI | 39 | MWA | 1 | cascination | FALSE | TRUE | TRUE | recULsp8CL03eFSK6 | CRC | 1 | 53 | recIC5HLb7UPaDSH0 | 23-05-18 | FALSE | None | 2 | 133 | 3764 | large | FALSE |
| 815 | 1 | SURGNOVA | IVb | 20 | MWA | 1 | cascination | TRUE | FALSE | FALSE | recurRH6MsQax3nQa | HCC | 1 | 72 | recP0T8vzmIHCDQ7g | 16-01-20 | FALSE | None | 1 | 63 | 704 | small | FALSE |
| 817 | 1 | Emprint | VIII | 18 | MWA | 1 | cascination | TRUE | FALSE | FALSE | recuTWkAxTX9Wm9l9 | HCC | 1 | 52 | reciKzCd6fRVSk9i3 | 18-10-18 | FALSE | None | 1 | 68 | 2543 | small | TRUE |
| 818 | 1 | Emprint | II | 13 | MWA | 1 | cascination | FALSE | FALSE | FALSE | recUVjHJgMgEEfvan | HCC | 1 | 59 | recFceFVC3nJQeRbP | 10-07-19 | FALSE | None | 3 | 106 | 1237 | small | FALSE |
| 819 | 1 | Emprint | VIII | 19 | MWA | 1 | cascination | TRUE | FALSE | FALSE | recUVjHJgMgEEfvan | HCC | 1 | 59 | recgcYCirdvxFs3Ao | 18-06-19 | TRUE | I | 2 | 213 | 3548 | small | TRUE |
| 820 | 1 | Emprint | II | 16 | MWA | 1 | cascination | FALSE | FALSE | TRUE | recUVjHJgMgEEfvan | HCC | 1 | 59 | recgcYCirdvxFs3Ao | 18-06-19 | TRUE | I | 2 | 213 | 3548 | small | FALSE |
| 821 | 1 | Emprint | VIII | 11 | MWA | 1 | cascination | TRUE | TRUE | FALSE | recUVjHJgMgEEfvan | HCC | 1 | 59 | recFceFVC3nJQeRbP | 10-07-19 | FALSE | None | 3 | 106 | 1237 | small | TRUE |
| 822 | 1 | Emprint | III | 14 | MWA | 1 | cascination | TRUE | TRUE | FALSE | recUVjHJgMgEEfvan | HCC | 1 | 59 | recFceFVC3nJQeRbP | 10-07-19 | FALSE | None | 3 | 106 | 1237 | small | FALSE |
| 826 | 1 | Emprint | VI | 11 | MWA | 1 | cascination | TRUE | FALSE | FALSE | recuvSAZkqtL1DLdX | CRC | 1 | 64 | recxkxqtkWRXEIeB8 | 17-11-17 | FALSE | None | 1 | 68 | 1780 | small | FALSE |
| 829 | 1 | TATO | VI | 13 | MWA | 1 | cascination | TRUE | FALSE | TRUE | recVd1HocAS6faD6o | HCC | 1 | 67 | rec99pTxcSDpiW3y8 | 21-08-19 | FALSE | None | 2 | 170 | 2348 | small | FALSE |
| 830 | 1 | TATO | VIII | 27 | MWA | 1 | cascination | TRUE | FALSE | FALSE | recVd1HocAS6faD6o | HCC | 1 | 67 | rec99pTxcSDpiW3y8 | 21-08-19 | FALSE | None | 2 | 170 | 2348 | small | TRUE |
| 831 | 1 | SURGNOVA | II | 15 | MWA | 1 | cascination | TRUE | TRUE | FALSE | recVd1HocAS6faD6o | HCC | 1 | 67 | rect43HVsW8jjFRQF | 11-03-20 | FALSE | None | 1 | 55 | 2442 | small | FALSE |
| 833 | 1 | Acculis | V | 15 | MWA | 1 | cascination | TRUE | FALSE | FALSE | recVFW4El0pbJ0WnH | HCC | 1 | 61 | recJwzmhUmjXFYWkB | 15-09-17 | FALSE | None | 1 | 90 | 2629 | small | FALSE |
| 841 | 1 | Acculis | VIII | 24 | MWA | 1 | cascination | FALSE | FALSE | FALSE | recvoofvFSwcT5ZOH | CCC | 1 | 72 | recGad9fB7K87ng3v | 18-03-20 | TRUE | III | 2 | 189 | 2422 | small | TRUE |
| 842 | 1 | Acculis | VIII | 11 | MWA | 1 | cascination | TRUE | FALSE | FALSE | recvoofvFSwcT5ZOH | CCC | 1 | 72 | recGad9fB7K87ng3v | 18-03-20 | TRUE | III | 2 | 189 | 2422 | small | TRUE |
| 843 | 1 | Acculis | V | 28 | MWA | 1 | cascination | FALSE | FALSE | FALSE | recvpqPinfzLcptPI | HCC | 1 | 61 | recjg37VWBtx8ntMC | 03-08-16 | FALSE | None | 1 | 44 | 3992 | small | FALSE |
| 844 | 1 | TATO | VII | 23 | MWA | 1 | cascination | FALSE | TRUE | FALSE | recVT337CiwODliFR | CRC | 1 | 71 | recxl2M4yUwFzdKG9 | 10-07-19 | FALSE | None | 3 | 211 | 2009 | small | TRUE |
| 846 | 1 | TATO | III | 18 | MWA | 1 | cascination | FALSE | FALSE | FALSE | recVT337CiwODliFR | CRC | 1 | 71 | recxl2M4yUwFzdKG9 | 10-07-19 | FALSE | None | 3 | 211 | 2009 | small | FALSE |
| 847 | 1 | Acculis | VII | 14 | MWA | 1 | cascination | FALSE | FALSE | FALSE | recw0fpXk4ADrH3Qg | HCC | 1 | 77 | recNRFyBXfx7TIPNE | 23-05-19 | FALSE | None | 3 | 114 | 2168 | small | TRUE |
| 848 | 1 | Acculis | IVa | 17 | MWA | 1 | cascination | TRUE | FALSE | FALSE | recw0fpXk4ADrH3Qg | HCC | 1 | 77 | recNRFyBXfx7TIPNE | 23-05-19 | FALSE | None | 3 | 114 | 2168 | small | FALSE |
| 849 | 1 | Acculis | III | 7 | MWA | 1 | cascination | FALSE | FALSE | FALSE | recw0fpXk4ADrH3Qg | HCC | 1 | 77 | recNRFyBXfx7TIPNE | 23-05-19 | FALSE | None | 3 | 114 | 2168 | small | FALSE |
| 863 | 1 | SURGNOVA | II | 8 | MWA | 1 | cascination | TRUE | FALSE | FALSE | recWJ7OeR3RzKBlGT | HCC | 1 | 80 | recrruWp4jSbwssQQ | 10-03-20 | FALSE | None | 1 | 103 | 2283 | small | FALSE |
| 868 | 0 | Emprint | IVa | 12 | MWA | 1 | cascination | FALSE | FALSE | FALSE | recWROJgC37Bumkyl | CRC | 0 | 60 | recKIr1Tbp1nqkkvf | 07-08-18 | TRUE | I | 1 | 73 | 1873 | small | FALSE |
| 869 | 1 | SURGNOVA | VI | 27 | MWA | 1 | cascination | TRUE | FALSE | FALSE | recwUtDzwpocvUvQK | HCC | 1 | 71 | recXY21A5t0gxWD9K | 05-03-20 | FALSE | None | 1 | 66 | 1135 | small | FALSE |
| 876 | 0 | large Spherical Ablation Zone-Reinforced Antenna | V | 13 | MWA | 1 | cascination | TRUE | FALSE | FALSE | recXaTMEag9XAxDGU | CRC | 0 | 64 | recpAVBg3aBcORwBF | 16-04-19 | FALSE | None | 3 | 121 | 1681 | small | FALSE |
| 877 | 0 | large Spherical Ablation Zone-Reinforced Antenna | V | 18 | MWA | 1 | cascination | FALSE | FALSE | TRUE | recXaTMEag9XAxDGU | CRC | 0 | 64 | recpAVBg3aBcORwBF | 16-04-19 | FALSE | None | 3 | 121 | 1681 | small | FALSE |
| 878 | 0 | Acculis | VI | 31 | MWA | 1 | cascination | TRUE | FALSE | FALSE | recxCinl0fz7HTohR | HCC | 0 | 79 | recpju5o9hTsIRahY | 14-07-15 | TRUE | I | 2 | 120 | 4078 | large | FALSE |
| 880 | 0 | Acculis | II | 20 | MWA | 1 | cascination | TRUE | FALSE | FALSE | recxCinl0fz7HTohR | HCC | 0 | 79 | recpju5o9hTsIRahY | 14-07-15 | TRUE | I | 2 | 120 | 4078 | small | FALSE |
| 888 | 1 | SURGNOVA | VIII | 19 | MWA | 1 | cascination | FALSE | TRUE | TRUE | recxdo1WtRL4HoIfn | HCC | 0 | 72 | recXiHUys7NZdsdDw | 29-01-20 | FALSE | None | 1 | 135 | 3227 | small | TRUE |
| 898 | 1 | Acculis | VIII | 14 | MWA | 1 | cascination | FALSE | FALSE | FALSE | recXUc7QjTY1RTvrb | CRC | 1 | 59 | recLLPptSfSNNRvo5 | 18-05-16 | FALSE | None | 2 | 51 | 3529 | small | TRUE |
| 900 | 1 | Acculis | VIII | 16 | MWA | 1 | cascination | FALSE | FALSE | TRUE | recXUc7QjTY1RTvrb | CRC | 1 | 59 | recLLPptSfSNNRvo5 | 18-05-16 | FALSE | None | 2 | 51 | 3529 | small | TRUE |
| 908 | 0 | Emprint | IVb | 23 | MWA | 1 | cascination | FALSE | TRUE | FALSE | recy87NGw1Za8MG1A | CCC | 1 | 78 | recHJose0HLCTP2ds | 08-03-19 | FALSE | None | 1 | 79 | 1546 | small | FALSE |
| 910 | 1 | Emprint | IVa | 16 | MWA | 1 | cascination | TRUE | TRUE | TRUE | recy9mJqFN2zT5Rtu | HCC | 1 | 77 | recF71RIMEwdWRd3P | 13-03-19 | FALSE | None | 3 | 205 | 3363 | small | FALSE |
| 911 | 1 | Emprint | VIII | 10 | MWA | 1 | cascination | FALSE | FALSE | TRUE | recy9mJqFN2zT5Rtu | HCC | 1 | 77 | recPwaY9QWzUES0an | 12-03-20 | FALSE | None | 1 | 48 | 1260 | small | TRUE |
| 912 | 1 | Emprint | II | 12 | MWA | 1 | cascination | TRUE | TRUE | FALSE | recy9mJqFN2zT5Rtu | HCC | 1 | 77 | recndPDp70AZkw3GN | 19-11-19 | FALSE | None | 1 | 74 | 2158 | small | FALSE |
| 914 | 0 | TATO | II | 11 | MWA | 1 | cascination | TRUE | TRUE | FALSE | recy9mJqFN2zT5Rtu | HCC | 1 | 77 | recgOIMi6UUVDIFH2 | 15-05-19 | FALSE | None | 1 | 57 | 1425 | small | FALSE |
| 915 | 0 | Emprint | II | 24 | MWA | 1 | cascination | TRUE | FALSE | FALSE | recy9mJqFN2zT5Rtu | HCC | 1 | 77 | recF71RIMEwdWRd3P | 13-03-19 | FALSE | None | 3 | 205 | 3363 | small | FALSE |
| 916 | 1 | Emprint | II | 18 | MWA | 1 | cascination | FALSE | FALSE | TRUE | recy9mJqFN2zT5Rtu | HCC | 1 | 77 | recF71RIMEwdWRd3P | 13-03-19 | FALSE | None | 3 | 205 | 3363 | small | FALSE |
| 917 | 0 | TATO | IVa | 28 | MWA | 1 | cascination | TRUE | FALSE | TRUE | recYddxMK6na0o0jl | CRC | 1 | 59 | recZvCRIsamd0XlCJ | 14-05-19 | FALSE | None | 2 | 138 | 2007 | small | FALSE |
| 918 | 0 | Emprint | IVb | 15 | MWA | 1 | cascination | TRUE | FALSE | FALSE | recYddxMK6na0o0jl | CRC | 1 | 59 | recwH4gfBDZzbRNCZ | 12-08-19 | FALSE | None | 2 | 122 | 3255 | small | FALSE |
| 920 | 1 | Emprint | II | 14 | MWA | 1 | cascination | TRUE | FALSE | TRUE | recYddxMK6na0o0jl | CRC | 1 | 59 | recwH4gfBDZzbRNCZ | 12-08-19 | FALSE | None | 2 | 122 | 3255 | small | FALSE |
| 921 | 0 | TATO | II | 24 | MWA | 1 | cascination | FALSE | TRUE | TRUE | recYddxMK6na0o0jl | CRC | 1 | 59 | recZvCRIsamd0XlCJ | 14-05-19 | FALSE | None | 2 | 138 | 2007 | small | FALSE |
| 927 | 1 | Emprint | II | 21 | MWA | 1 | cascination | FALSE | FALSE | FALSE | recyGfePTn0aIskTy | Other | 1 | 69 | rechnxJeZebsBofZ1 | 11-02-20 | TRUE | I | 1 | 64 | 2232 | small | FALSE |
| 932 | 1 | TATO | IVa | 15 | MWA | 1 | cascination | TRUE | FALSE | FALSE | recYltUMJvXUq7ORz | HCC | 1 | 62 | rec9miVfmjKWQ2gFx | 20-03-19 | FALSE | None | 1 | 160 | 4836 | small | FALSE |
| 951 | 1 | TATO | IVb | 11 | MWA | 1 | cascination | TRUE | FALSE | TRUE | recZBcCqvpKPHmGnn | HCC | 1 | 43 | recwLZpHO6eF86ilp | 07-04-20 | FALSE | None | 1 | 145 | 2607 | small | FALSE |
| 957 | 1 | Emprint | VI | 13 | MWA | 1 | cascination | TRUE | FALSE | FALSE | recZsHXEiyZyHWYIj | HCC | 1 | 57 | recNjkfhRUTkDUYFd | 14-04-16 | FALSE | None | 1 | 57 | 1945 | small | FALSE |
| 963 | 1 | TATO | IVb | 25 | MWA | 1 | cascination | TRUE | FALSE | FALSE | reczXVzT6IzoiLfSF | Other | 1 | 61 | recMiAak1glS0Plr4 | 24-04-19 | FALSE | None | 2 | 155 | 2633 | small | FALSE |
| 964 | 0 | TATO | VI | 35 | MWA | 1 | cascination | FALSE | TRUE | FALSE | reczXVzT6IzoiLfSF | Other | 1 | 61 | recMiAak1glS0Plr4 | 24-04-19 | FALSE | None | 2 | 155 | 2633 | large | FALSE |
| 965 | 1 | Emprint | VIII | 18 | MWA | 1 | cascination | TRUE | TRUE | TRUE | reczXVzT6IzoiLfSF | Other | 1 | 61 | recOlWdXllAkpRyvF | 21-08-19 | FALSE | None | 1 | 80 | 2794 | small | TRUE |
| 57 | 1 | SURGNOVA | VII | 23 | MWA | 1 | cascination freehand | TRUE | FALSE | TRUE | rec4qiVxbUnVpTbzQ | HCC | 1 | 65 | reckezHXIsAvMvla6 | 31-03-20 | FALSE | None | 1 | 82 | 2367 | small | TRUE |
| 58 | 0 | Emprint | VII | 23 | MWA | 1 | cascination freehand | TRUE | FALSE | FALSE | rec4qiVxbUnVpTbzQ | HCC | 1 | 65 | rec3icJkSuAI3xpxa | 19-09-18 | FALSE | None | 1 | 88 | 1429 | small | TRUE |
| 116 | 0 | Emprint | VII | 3 | MWA | 1 | cascination freehand | TRUE | FALSE | FALSE | rec7zM4muLOSYdjvo | Other | 0 | 42 | rec7kf98s3tFBI8dr | 28-07-16 | FALSE | None | 2 | 100 | 1716 | small | TRUE |
| 118 | 0 | Emprint | VII | 6 | MWA | 1 | cascination freehand | FALSE | FALSE | FALSE | rec7zM4muLOSYdjvo | Other | 0 | 42 | rec7kf98s3tFBI8dr | 28-07-16 | FALSE | None | 2 | 100 | 1716 | small | TRUE |
| 175 | 1 | Emprint | III | 20 | MWA | 1 | cascination freehand | FALSE | FALSE | FALSE | recaGRboGDhvQg4oe | HCC | 1 | 83 | recD21S8W9vPKKIc3 | 17-01-19 | FALSE | None | 4 | 104 | 2616 | small | FALSE |
| 630 | 1 | Emprint | VI | 9 | MWA | 1 | cascination freehand | FALSE | FALSE | FALSE | recoiBHEOIDMsNIRm | Other | 1 | 75 | recz4Vh7B3KMNPC2B | 02-02-17 | FALSE | None | 1 | 56 | 2684 | small | FALSE |
| 798 | 1 | Acculis | VII | 16 | MWA | 1 | cascination freehand | TRUE | TRUE | FALSE | recUcIoPaHtOpPye1 | HCC | 1 | 65 | recI3lGsJ3nAlNybV | 17-11-16 | FALSE | None | 1 | 58 | 1646 | small | TRUE |
| 6 | 1 | Emprint | IVb | 28 | MWA | 0 | fluoroskopie | TRUE | TRUE | TRUE | rec14EgS97RxMIzPu | CRC | 1 | 43 | recPVhyvItLjIGzMo | 14-03-14 | FALSE | None | 1 | 136 | 2513 | small | FALSE |
| 12 | 1 | Emprint | VI | 25 | MWA | 0 | fluoroskopie | FALSE | FALSE | TRUE | rec1dPUUWMfPoDqH8 | HCC | 1 | 79 | recP4scxv89BkBqE2 | 22-02-18 | FALSE | None | 1 | 62 | 2377 | small | FALSE |
| 25 | 1 | Emprint | VII | 8 | MWA | 0 | fluoroskopie | FALSE | FALSE | FALSE | rec28si3RjlGi9T0t | HCC | 1 | 72 | recQZ5AGqFfse7TXn | 27-01-16 | FALSE | None | 1 | 87 | 2114 | small | TRUE |
| 26 | 1 | Emprint | V | 10 | MWA | 0 | fluoroskopie | FALSE | FALSE | FALSE | rec28si3RjlGi9T0t | HCC | 1 | 72 | reckGkjPVBKZ9fYOV | 13-08-15 | FALSE | None | 2 | 107 | 2953 | small | FALSE |
| 27 | 1 | Emprint | VIII | 11 | MWA | 0 | fluoroskopie | TRUE | TRUE | FALSE | rec28si3RjlGi9T0t | HCC | 1 | 72 | reckGkjPVBKZ9fYOV | 13-08-15 | FALSE | None | 2 | 107 | 2953 | small | TRUE |
| 32 | 0 | Acculis | II | 5 | MWA | 0 | fluoroskopie | FALSE | FALSE | FALSE | rec2le7fzUjZBVtPG | Other | 0 | 66 | recz5oAR1L42sKDIM | 08-04-14 | FALSE | None | 2 | 280 |  | small | FALSE |
| 33 | 1 | Acculis | VI | 7 | MWA | 0 | fluoroskopie | FALSE | FALSE | FALSE | rec2le7fzUjZBVtPG | Other | 0 | 66 | recz5oAR1L42sKDIM | 08-04-14 | FALSE | None | 2 | 280 |  | small | FALSE |
| 44 | 1 | Emprint | VI | 12 | MWA | 0 | fluoroskopie | TRUE | FALSE | TRUE | rec45PrnN2qSe7rzS | HCC | 0 | 52 | recSWsJ0mokEa5rwM | 20-05-16 | FALSE | None | 1 | 207 | 4689 | small | FALSE |
| 50 | 1 | Acculis | VIII | 30 | MWA | 0 | fluoroskopie | FALSE | FALSE | FALSE | rec4e7vE4wquiRvBi | HCC | 1 | 58 | rec2cNRzl4LuvCqbw | 07-04-16 | FALSE | None | 2 | 107 | 3372 | small | TRUE |
| 63 | 1 | Acculis | VI | 56 | MWA | 0 | fluoroskopie | TRUE | FALSE | FALSE | rec4S8WQGfZWp1pt9 | HCC | 1 | 50 | recSJLetfBTIlZpq3 | 02-02-16 | FALSE | None | 1 | 101 | 1685 | large | FALSE |
| 75 | 1 | Emprint | V | 10 | MWA | 0 | fluoroskopie | TRUE | TRUE | FALSE | rec6ai6zL94cIw52C | Other | 0 | 39 | recU1VockvYYEu5Zw | 13-11-18 | FALSE | None | 1 |  | 695 | small | FALSE |
| 76 | 0 | Acculis | VIII | 43 | MWA | 0 | fluoroskopie | FALSE | FALSE | TRUE | rec6atAlyJSWDNi2o | HCC | 1 | 71 | recJKsy7WrrDMGzTP | 19-10-16 | FALSE | None | 1 | 77 | 1629 | large | TRUE |
| 80 | 1 | Acculis | III | 32 | MWA | 0 | fluoroskopie | FALSE | FALSE | FALSE | rec6B5RcuFBX5Hy4T | HCC | 1 | 63 | rec6tJFnLKeuYEPcw | 27-10-17 | FALSE | None | 1 | 144 | 3158 | large | FALSE |
| 81 | 1 | Emprint | IVa | 15 | MWA | 0 | fluoroskopie | TRUE | FALSE | TRUE | rec6B5RcuFBX5Hy4T | HCC | 1 | 63 | recUsI9P31vJ1Fy1N | 30-01-18 | FALSE | None | 1 | 43 | 2067 | small | FALSE |
| 87 | 1 | Acculis | VIII | 15 | MWA | 0 | fluoroskopie | TRUE | TRUE | FALSE | rec6SsZq5DMJlZo2r | Other | 1 | 68 | recUJ5h3EZGvhXoZl | 06-11-14 | FALSE | None | 2 | 85 | 2438 | small | TRUE |
| 89 | 1 | Acculis | VIII | 7 | MWA | 0 | fluoroskopie | TRUE | TRUE | FALSE | rec6SsZq5DMJlZo2r | Other | 1 | 68 | recUJ5h3EZGvhXoZl | 06-11-14 | FALSE | None | 2 | 85 | 2438 | small | TRUE |
| 93 | 1 | Emprint | VII | 21 | MWA | 0 | fluoroskopie | TRUE | FALSE | FALSE | rec6Ufq4m1L5BvybE | HCC | 1 | 47 | recwfIWyNJBHkecZ3 | 06-10-17 | FALSE | None | 1 | 80 | 2724 | small | TRUE |
| 102 | 1 | Emprint | V | 10 | MWA | 0 | fluoroskopie | TRUE | FALSE | FALSE | rec7MbQEwNpoUw0K0 | HCC | 1 | 52 | recxynadfOpxcyXja | 20-06-18 | FALSE | None | 2 | 96 | 1904 | small | FALSE |
| 107 | 1 | Emprint | IVb | 15 | MWA | 0 | fluoroskopie | FALSE | FALSE | FALSE | rec7MbQEwNpoUw0K0 | HCC | 1 | 52 | recxynadfOpxcyXja | 20-06-18 | FALSE | None | 2 | 96 | 1904 | small | FALSE |
| 121 | 1 | Emprint | VI | 13 | MWA | 0 | fluoroskopie | TRUE | FALSE | FALSE | rec8I6UiuRn28yN5I | HCC | 1 | 65 | recWzJcV3dhO4wN2C | 28-04-16 | FALSE | None | 4 | 127 | 3486 | small | FALSE |
| 123 | 1 | Acculis | VI | 30 | MWA | 0 | fluoroskopie | TRUE | FALSE | FALSE | rec8I6UiuRn28yN5I | HCC | 1 | 65 | recLTzWmXx0iiD2ro | 06-12-16 | TRUE | IV | 1 | 42 | 2139 | small | FALSE |
| 126 | 1 | Acculis | II | 35 | MWA | 0 | fluoroskopie | TRUE | FALSE | FALSE | rec8Pwru2Ri3y7c5e | HCC | 1 | 63 | recWG9J7BdcPu5c28 | 18-05-18 | FALSE | None | 1 | 67 | 4500 | large | FALSE |
| 138 | 1 | Acculis | VIII | 32 | MWA | 0 | fluoroskopie | TRUE | TRUE | FALSE | rec93cpKjDleORQwU | HCC | 1 | 59 | recXUPHnSZf0KPQtO | 06-02-15 | TRUE | IV | 1 | 101 | 2920 | large | TRUE |
| 146 | 1 | Acculis | VIII | 6 | MWA | 0 | fluoroskopie | TRUE | TRUE | FALSE | rec9EOqUDugfqnJGV | HCC | 1 | 69 | receEf18LgdJuhGC5 | 18-05-16 | FALSE | None | 4 | 70 | 2972 | small | TRUE |
| 150 | 1 | Acculis | V | 17 | MWA | 0 | fluoroskopie | TRUE | FALSE | FALSE | rec9FINNe7oyHYp5u | CRC | 1 | 53 | reccIKT7268uosImS | 16-01-14 | FALSE | None | 4 | 75 | 2716 | small | FALSE |
| 154 | 0 | Acculis | II | 13 | MWA | 0 | fluoroskopie | FALSE | FALSE | FALSE | rec9FINNe7oyHYp5u | CRC | 1 | 53 | reccIKT7268uosImS | 16-01-14 | FALSE | None | 4 | 75 | 2716 | small | FALSE |
| 155 | 1 | Acculis | IVa | 13 | MWA | 0 | fluoroskopie | TRUE | TRUE | FALSE | rec9FINNe7oyHYp5u | CRC | 1 | 53 | reccIKT7268uosImS | 16-01-14 | FALSE | None | 4 | 75 | 2716 | small | FALSE |
| 156 | 1 | Acculis | VIII | 18 | MWA | 0 | fluoroskopie | TRUE | TRUE | FALSE | rec9FINNe7oyHYp5u | CRC | 1 | 53 | reccIKT7268uosImS | 16-01-14 | FALSE | None | 4 | 75 | 2716 | small | TRUE |
| 161 | 1 | Acculis | VII | 36 | MWA | 0 | fluoroskopie | TRUE | FALSE | TRUE | rec9Tsk4zPIHc4Fhd | Other | 1 | 70 | recXK5CH8bCt82Fe7 | 27-10-17 | FALSE | None | 1 | 75 | 1991 | large | TRUE |
| 171 | 0 | Emprint | VII | 13 | MWA | 0 | fluoroskopie | FALSE | FALSE | FALSE | recaDNv6jgTAmHhNk | HCC | 1 | 51 | recZrqa7OdQUUbEPa | 17-08-16 | FALSE | None | 1 |  | 2142 | small | TRUE |
| 185 | 1 | Acculis | II | 14 | MWA | 0 | fluoroskopie | TRUE | TRUE | FALSE | recaQ6WQd4I6UrwWU | CRC | 1 | 48 | recYHJetMqCSQpwTO | 04-03-14 | FALSE | None | 2 | 354 |  | small | FALSE |
| 187 | 1 | Acculis | IVb | 21 | MWA | 0 | fluoroskopie | TRUE | FALSE | FALSE | recaQ6WQd4I6UrwWU | CRC | 1 | 48 | rec3CWQXut2IMabLr | 27-01-14 | FALSE | None | 2 | 67 | 3935 | small | FALSE |
| 188 | 1 | Acculis | II | 18 | MWA | 0 | fluoroskopie | FALSE | FALSE | FALSE | recaQ6WQd4I6UrwWU | CRC | 1 | 48 | rec3CWQXut2IMabLr | 27-01-14 | FALSE | None | 2 | 67 | 3935 | small | FALSE |
| 189 | 1 | Emprint | III | 16 | MWA | 0 | fluoroskopie | TRUE | FALSE | FALSE | recAYFnCDfvu8PWeI | CRC | 1 | 55 | recoPiFfcBpg4NWbC | 13-07-18 | FALSE | None | 2 | 55 | 1135 | small | FALSE |
| 190 | 0 | Emprint | IVb | 14 | MWA | 0 | fluoroskopie | TRUE | TRUE | TRUE | recAYFnCDfvu8PWeI | CRC | 1 | 55 | recoPiFfcBpg4NWbC | 13-07-18 | FALSE | None | 2 | 55 | 1135 | small | FALSE |
| 202 | 0 | Emprint | II | 29 | MWA | 0 | fluoroskopie | FALSE | FALSE | TRUE | recBMn48U4emdQyPS | HCC | 1 | 59 | recy2gS3eBAL1lNzY | 16-03-17 | FALSE | None | 1 | 85 | 3553 | small | FALSE |
| 206 | 1 | Acculis | IVa | 24 | MWA | 0 | fluoroskopie | TRUE | FALSE | FALSE | recbno7julSxBd6wd | HCC | 1 | 63 | recrquiJCGa6vwMXc | 15-12-16 | FALSE | None | 1 | 33 | 1530 | small | FALSE |
| 251 | 0 | Acculis | II | 45 | MWA | 0 | fluoroskopie | TRUE | TRUE | FALSE | recCrIPhYsjGKjB2R | HCC | 1 | 69 | recqil7UxOdsGhBZL | 20-05-14 | FALSE | None | 1 | 116 | 5113 | large | FALSE |
| 253 | 1 | Emprint | VI | 18 | MWA | 0 | fluoroskopie | TRUE | FALSE | FALSE | reccURK9BxTU1ygcE | Other | 1 | 84 | recmBLqWlPt1Z36Y0 | 18-09-14 | FALSE | None | 2 | 81 | 2383 | small | FALSE |
| 272 | 1 | Acculis | VI | 8 | MWA | 0 | fluoroskopie | TRUE | FALSE | FALSE | recdJDeuQP340LUhW | HCC | 1 | 72 | rec1Agw7pbXQWJUeQ | 13-03-13 | FALSE | None | 1 | 144 | 2166 | small | FALSE |
| 296 | 1 | Acculis | IVb | 50 | MWA | 0 | fluoroskopie | FALSE | FALSE | TRUE | recEbg0ADfDF4LCyT | HCC | 1 | 61 | recs2TidcBxr0JCvN | 12-07-17 | FALSE | None | 1 | 70 | 3906 | large | FALSE |
| 301 | 0 | Emprint | IVa | 22 | MWA | 0 | fluoroskopie | TRUE | FALSE | FALSE | recENyOGrWXGdQAva | CCC | 0 | 77 | recsEb6j0iRs9OAs4 | 20-04-16 | FALSE | None | 1 | 119 | 3166 | small | FALSE |
| 306 | 1 | Acculis | VIII | 14 | MWA | 0 | fluoroskopie | TRUE | TRUE | FALSE | recEw0oUIiHWEBKja | HCC | 0 | 59 | recsnDGxhEBIAzKg4 | 27-02-14 | FALSE | None | 1 | 102 |  | small | TRUE |
| 307 | 0 | Acculis | VIII | 20 | MWA | 0 | fluoroskopie | TRUE | TRUE | FALSE | recEw0oUIiHWEBKja | HCC | 0 | 59 | recTD4oxtvKXDZ7lT | 16-01-14 | FALSE | None | 1 | 66 | 1936 | small | TRUE |
| 337 | 1 | Emprint | IVb | 11 | MWA | 0 | fluoroskopie | TRUE | TRUE | TRUE | recG20xZ19ZCluhKf | HCC | 1 | 57 | reckvf1TKGWTXLMFt | 11-07-17 | FALSE | None | 5 | 152 | 6052 | small | FALSE |
| 338 | 1 | Emprint | VII | 23 | MWA | 0 | fluoroskopie | TRUE | TRUE | FALSE | recG20xZ19ZCluhKf | HCC | 1 | 57 | recpeyw4019vUx9Oh | 11-08-15 | FALSE | None | 2 | 101 | 3881 | small | TRUE |
| 339 | 1 | Emprint | VII | 10 | MWA | 0 | fluoroskopie | TRUE | TRUE | FALSE | recG20xZ19ZCluhKf | HCC | 1 | 57 | recpeyw4019vUx9Oh | 11-08-15 | FALSE | None | 2 | 101 | 3881 | small | TRUE |
| 341 | 1 | Emprint | VI | 10 | MWA | 0 | fluoroskopie | TRUE | FALSE | FALSE | recG20xZ19ZCluhKf | HCC | 1 | 57 | reckvf1TKGWTXLMFt | 11-07-17 | FALSE | None | 5 | 152 | 6052 | small | FALSE |
| 343 | 1 | Emprint | III | 7 | MWA | 0 | fluoroskopie | FALSE | FALSE | FALSE | recG20xZ19ZCluhKf | HCC | 1 | 57 | reckvf1TKGWTXLMFt | 11-07-17 | FALSE | None | 5 | 152 | 6052 | small | FALSE |
| 345 | 1 | Emprint | IVa | 10 | MWA | 0 | fluoroskopie | FALSE | FALSE | FALSE | recG20xZ19ZCluhKf | HCC | 1 | 57 | reckvf1TKGWTXLMFt | 11-07-17 | FALSE | None | 5 | 152 | 6052 | small | FALSE |
| 346 | 1 | Emprint | VI | 15 | MWA | 0 | fluoroskopie | TRUE | FALSE | FALSE | recG20xZ19ZCluhKf | HCC | 1 | 57 | recuTDPCAvTohshH9 | 07-03-18 | FALSE | None | 4 | 157 | 8647 | small | FALSE |
| 347 | 1 | Emprint | III | 12 | MWA | 0 | fluoroskopie | TRUE | TRUE | FALSE | recG20xZ19ZCluhKf | HCC | 1 | 57 | reckvf1TKGWTXLMFt | 11-07-17 | FALSE | None | 5 | 152 | 6052 | small | FALSE |
| 348 | 1 | Emprint | III | 8 | MWA | 0 | fluoroskopie | FALSE | FALSE | FALSE | recG20xZ19ZCluhKf | HCC | 1 | 57 | recuTDPCAvTohshH9 | 07-03-18 | FALSE | None | 4 | 157 | 8647 | small | FALSE |
| 349 | 1 | Emprint | III | 7 | MWA | 0 | fluoroskopie | TRUE | FALSE | TRUE | recG20xZ19ZCluhKf | HCC | 1 | 57 | recuTDPCAvTohshH9 | 07-03-18 | FALSE | None | 4 | 157 | 8647 | small | FALSE |
| 351 | 1 | Emprint | III | 7 | MWA | 0 | fluoroskopie | TRUE | FALSE | TRUE | recG20xZ19ZCluhKf | HCC | 1 | 57 | recuTDPCAvTohshH9 | 07-03-18 | FALSE | None | 4 | 157 | 8647 | small | FALSE |
| 358 | 1 | Acculis | VIII | 27 | MWA | 0 | fluoroskopie | FALSE | FALSE | FALSE | recGhBH88VFWY9xcG | HCC | 1 | 67 | recu8eZLHhzIU7x9A | 23-11-16 | FALSE | None | 1 | 59 | 2216 | small | TRUE |
| 399 | 1 | Acculis | II | 5 | MWA | 0 | fluoroskopie | TRUE | FALSE | FALSE | rechFDj7TTrH1EoLg | HCC | 0 | 72 | rec5wgBKsfltXCoIa | 25-03-15 | TRUE | IV | 2 | 123 | 2584 | small | FALSE |
| 400 | 1 | Acculis | VIII | 16 | MWA | 0 | fluoroskopie | FALSE | FALSE | FALSE | rechFDj7TTrH1EoLg | HCC | 0 | 72 | rec5wgBKsfltXCoIa | 25-03-15 | TRUE | IV | 2 | 123 | 2584 | small | TRUE |
| 411 | 1 | Acculis | VII | 24 | MWA | 0 | fluoroskopie | TRUE | TRUE | FALSE | recHQ8LYkmjCeULnn | HCC | 1 | 76 | recvHL3BTIdoaSLkh | 17-06-15 | FALSE | None | 2 | 305 | 4554 | small | TRUE |
| 430 | 1 | Acculis | IVa | 14 | MWA | 0 | fluoroskopie | TRUE | TRUE | FALSE | recijuMFUHlP4NwyJ | HCC | 1 | 69 | rec6a74it3fB0LwvD | 15-01-14 | FALSE | None | 1 | 112 | 3214 | small | FALSE |
| 433 | 1 | Emprint | VIII | 18 | MWA | 0 | fluoroskopie | TRUE | FALSE | FALSE | recIkuylHNjfYkN9V | HCC | 1 | 64 | recwb7QYg9d1UiN6P | 08-02-11 | FALSE | None | 1 | 73 | 834 | small | TRUE |
| 448 | 1 | Emprint | V | 23 | MWA | 0 | fluoroskopie | FALSE | FALSE | FALSE | recjGDA74dIRTKGqT | HCC | 1 | 59 | reccKIKYVs6mNlalr | 20-11-18 | FALSE | None | 1 | 57 | 1101 | small | FALSE |
| 453 | 1 | Emprint | VI | 18 | MWA | 0 | fluoroskopie | TRUE | FALSE | FALSE | recJIJjtTYZcjUiaH | HCC | 0 | 66 | recxzmB6skTYfSi7B | 02-03-18 | FALSE | None | 1 | 57 | 1845 | small | FALSE |
| 457 | 0 | Acculis | VIII | 18 | MWA | 0 | fluoroskopie | TRUE | FALSE | FALSE | recJKYWq9BaNtQzds | CRC | 1 | 72 | recxBBe3IX4zpOzam | 31-05-12 | FALSE | None | 3 | 193 | 4162 | small | TRUE |
| 473 | 1 | Acculis | IVa | 30 | MWA | 0 | fluoroskopie | TRUE | TRUE | FALSE | recjtxDSm4N3WAR4n | CRC | 0 | 71 | rec7kaVvVqHPSyR1h | 21-11-16 | FALSE | None | 1 | 84 | 2844 | small | FALSE |
| 492 | 1 | Acculis | IVb | 23 | MWA | 0 | fluoroskopie | TRUE | FALSE | FALSE | reckDkmU3OpAPpLmz | HCC | 1 | 63 | rec8uXExCajmLnLjt | 23-03-15 | FALSE | None | 1 | 72 | 2586 | small | FALSE |
| 499 | 0 | Emprint | VI | 13 | MWA | 0 | fluoroskopie | FALSE | FALSE | TRUE | recKJvGaaLggczHvR | HCC | 1 | 73 | recKaMx4RU31dC1Jp | 03-11-16 | FALSE | None | 1 | 58 | 2159 | small | FALSE |
| 506 | 0 | Emprint | I | 16 | MWA | 0 | fluoroskopie | TRUE | FALSE | FALSE | recKNB1xSeAfTI0YY | HCC | 1 | 72 | rec8pJ8fzsdoXBkeJ | 04-12-15 | FALSE | None | 1 | 97 | 5382 | small | TRUE |
| 519 | 1 | Emprint | VIII | 24 | MWA | 0 | fluoroskopie | FALSE | FALSE | TRUE | recKT1QiZOQgxxA3N | HCC | 1 | 54 | recyKE8VyaK2tvA0H | 06-10-15 | FALSE | None | 1 | 112 | 4169 | small | TRUE |
| 520 | 1 | Acculis | I | 17 | MWA | 0 | fluoroskopie | TRUE | TRUE | TRUE | recKuRxYu7xGw7tfE | HCC | 0 | 52 | recyluPB3trss5tcy | 20-04-17 | FALSE | None | 1 | 95 | 1528 | small | TRUE |
| 528 | 0 | Acculis | III | 29 | MWA | 0 | fluoroskopie | TRUE | FALSE | FALSE | recKzpNVpj4dwzDhs | HCC | 0 | 59 | recEIHcUO8Mn6Z2Xd | 13-12-13 | FALSE | None | 1 | 85 | 2547 | small | FALSE |
| 539 | 1 | Emprint | IVa | 26 | MWA | 0 | fluoroskopie | TRUE | FALSE | FALSE | reclljqTsxRWYHtdg | CCC | 0 | 53 | rec9cWIw1TLIUFtaa | 17-01-17 | FALSE | None | 2 | 104 | 3352 | small | FALSE |
| 540 | 1 | Acculis | IVa | 25 | MWA | 0 | fluoroskopie | FALSE | FALSE | TRUE | reclljqTsxRWYHtdg | CCC | 0 | 53 | rec9cWIw1TLIUFtaa | 17-01-17 | FALSE | None | 2 | 104 | 3352 | small | FALSE |
| 544 | 0 | Acculis | IVa | 33 | MWA | 0 | fluoroskopie | FALSE | FALSE | TRUE | reclrH0xuZFGjnRvH | HCC | 1 | 79 | rec9ikia3lzsflRsB | 22-11-13 | FALSE | None | 1 | 40 | 1899 | large | FALSE |
| 552 | 0 | Emprint | VIII | 16 | MWA | 0 | fluoroskopie | TRUE | FALSE | FALSE | recM8MOxg0G88h6D7 | HCC | 1 | 59 | recZAmlxY9gdbbMUG | 13-11-17 | TRUE | I | 1 | 117 | 3197 | small | TRUE |
| 562 | 1 | Emprint | IVa | 25 | MWA | 0 | fluoroskopie | TRUE | TRUE | FALSE | recMPDbFFiR24lokQ | HCC | 0 | 75 | recAGgtieELO0johK | 14-11-18 | FALSE | None | 1 | 73 | 2023 | small | FALSE |
| 566 | 0 | Acculis | VIII | 45 | MWA | 0 | fluoroskopie | TRUE | TRUE | FALSE | recMuO3xWpVFqoQjF | HCC | 1 | 74 | recAlrlavLPrmmQgz | 17-05-13 | TRUE | II | 3 | 86 | 2815 | large | TRUE |
| 567 | 0 | Acculis | V | 17 | MWA | 0 | fluoroskopie | TRUE | FALSE | FALSE | recMuO3xWpVFqoQjF | HCC | 1 | 74 | recAlrlavLPrmmQgz | 17-05-13 | TRUE | II | 3 | 86 | 2815 | small | FALSE |
| 568 | 1 | Acculis | III | 20 | MWA | 0 | fluoroskopie | TRUE | FALSE | FALSE | recMuO3xWpVFqoQjF | HCC | 1 | 74 | recAlrlavLPrmmQgz | 17-05-13 | TRUE | II | 3 | 86 | 2815 | small | FALSE |
| 571 | 0 | Emprint | V | 8 | MWA | 0 | fluoroskopie | TRUE | FALSE | FALSE | recna0JggFfNC6PdW | Other | 1 | 59 | recb1D1TP19zy4PaQ | 11-03-16 | FALSE | None | 3 | 69 | 2372 | small | FALSE |
| 572 | 1 | Emprint | VI | 13 | MWA | 0 | fluoroskopie | TRUE | FALSE | FALSE | recna0JggFfNC6PdW | Other | 1 | 59 | recb1D1TP19zy4PaQ | 11-03-16 | FALSE | None | 3 | 69 | 2372 | small | FALSE |
| 573 | 1 | Emprint | V | 6 | MWA | 0 | fluoroskopie | TRUE | FALSE | FALSE | recna0JggFfNC6PdW | Other | 1 | 59 | recb1D1TP19zy4PaQ | 11-03-16 | FALSE | None | 3 | 69 | 2372 | small | FALSE |
| 585 | 0 | Acculis | III | 20 | MWA | 0 | fluoroskopie | TRUE | FALSE | FALSE | recNImgNI3mzvPxHz | HCC | 1 | 56 | recBzZyqhpglrNxEt | 09-03-16 | FALSE | None | 1 | 63 | 1678 | small | FALSE |
| 588 | 1 | Emprint | VI | 11 | MWA | 0 | fluoroskopie | FALSE | FALSE | TRUE | recNjPejqJN16ODDg | CRC | 1 | 64 | recBaswWZ5HN2MDAa | 04-07-18 | FALSE | None | 2 | 96 | 1419 | small | FALSE |
| 599 | 1 | Acculis | III | 11 | MWA | 0 | fluoroskopie | FALSE | FALSE | FALSE | recnL2Foy5eJtQiHJ | HCC | 1 | 56 | rectNUwdv7CNme5xj | 22-11-16 | FALSE | None | 6 | 85 | 3374 | small | FALSE |
| 603 | 1 | Emprint | VI | 19 | MWA | 0 | fluoroskopie | FALSE | TRUE | FALSE | recnp1snDwUs2tsvd | CCC | 1 | 77 | rec6ps3B6WG3HVfVL | 07-05-15 | FALSE | None | 1 | 66 | 1901 | small | FALSE |
| 605 | 1 | Acculis | VII | 11 | MWA | 0 | fluoroskopie | TRUE | TRUE | FALSE | recNShHY38zqUk55k | HCC | 1 | 61 | recBJUZBCutcQi52e | 06-03-14 | FALSE | None | 1 | 112 | 5560 | small | TRUE |
| 618 | 0 | Acculis | VII | 18 | MWA | 0 | fluoroskopie | TRUE | TRUE | FALSE | reco08mvtSxoHww2B | HCC | 1 | 75 | receZnyxoxhxj3tFr | 27-11-13 | FALSE | None | 1 | 37 | 942 | small | TRUE |
| 632 | 1 | Emprint | IVb | 6 | MWA | 0 | fluoroskopie | TRUE | FALSE | FALSE | recoiBHEOIDMsNIRm | Other | 1 | 75 | recSpr4F84hxJulMO | 07-12-16 | FALSE | None | 1 | 41 | 1978 | small | FALSE |
| 637 | 1 | Emprint | IVa | 11 | MWA | 0 | fluoroskopie | TRUE | TRUE | FALSE | recOuKFeuXNHVKVE1 | HCC | 1 | 77 | recKN9lBOlk4xeMha | 02-03-18 | FALSE | None | 2 | 41 | 1802 | small | FALSE |
| 638 | 1 | Emprint | IVa | 14 | MWA | 0 | fluoroskopie | TRUE | TRUE | FALSE | recOuKFeuXNHVKVE1 | HCC | 1 | 77 | recKN9lBOlk4xeMha | 02-03-18 | FALSE | None | 2 | 41 | 1802 | small | FALSE |
| 660 | 1 | Emprint | III | 34 | MWA | 0 | fluoroskopie | TRUE | FALSE | FALSE | recpg0de5hvOLChde | HCC | 1 | 52 | recd7DvREDpAHAha8 | 27-04-16 | FALSE | None | 3 | 314 | 9969 | large | FALSE |
| 688 | 1 | Acculis | VIII | 8 | MWA | 0 | fluoroskopie | FALSE | FALSE | FALSE | recQbXZN5oTNPSXg8 | CRC | 0 | 55 | recE2AhqEKNzLQXd2 | 25-09-14 | FALSE | None | 1 | 35 |  | small | TRUE |
| 693 | 0 | Emprint | V | 42 | MWA | 0 | fluoroskopie | TRUE | TRUE | TRUE | recqhNLArfuPj7C46 | HCC | 1 | 82 | recwgt04ji83O1TzT | 11-01-17 | FALSE | None | 1 | 67 | 2799 | large | FALSE |
| 694 | 1 | Acculis | VIII | 14 | MWA | 0 | fluoroskopie | TRUE | TRUE | FALSE | recqoETJuTmqocXA5 | HCC | 1 | 71 | recefhbm3fgckaXxZ | 16-03-16 | FALSE | None | 2 | 97 | 1959 | small | TRUE |
| 695 | 1 | Acculis | IVb | 8 | MWA | 0 | fluoroskopie | FALSE | FALSE | TRUE | recqoETJuTmqocXA5 | HCC | 1 | 71 | recefhbm3fgckaXxZ | 16-03-16 | FALSE | None | 2 | 97 | 1959 | small | FALSE |
| 698 | 1 | Acculis | VI | 17 | MWA | 0 | fluoroskopie | FALSE | FALSE | FALSE | recQQwNfz0g7UWAqP | HCC | 1 | 67 | recEH95S8maTQUAnJ | 15-12-15 | TRUE | III | 2 | 178 | 4067 | small | FALSE |
| 699 | 1 | Acculis | VIII | 43 | MWA | 0 | fluoroskopie | TRUE | FALSE | FALSE | recQRd8oYU0RiWetP | HCC | 1 | 81 | recEIQq1xgUDeUeqJ | 06-12-17 | FALSE | None | 1 | 218 | 4143 | large | TRUE |
| 701 | 1 | Acculis | IVa | 11 | MWA | 0 | fluoroskopie | TRUE | TRUE | TRUE | recQSHfTa5cdsW8gM | CRC | 1 | 54 | rec1ZskPXftFN9Y20 | 23-11-16 | FALSE | None | 1 | 90 | 2308 | small | FALSE |
| 704 | 1 | Acculis | VI | 37 | MWA | 0 | fluoroskopie | TRUE | FALSE | FALSE | recqTqs8NGt9va51r | HCC | 0 | 65 | receK3KLm2nVr85Yl | 31-03-15 | TRUE | I | 1 | 104 | 3354 | large | FALSE |
| 723 | 1 | Acculis | V | 4 | MWA | 0 | fluoroskopie | FALSE | FALSE | FALSE | recrP6zoUcvLVdEh7 | Other | 0 | 69 | recfGJR1typxRbEe1 | 29-10-13 | FALSE | None | 2 | 170 | 3344 | small | FALSE |
| 725 | 1 | Emprint | VII | 12 | MWA | 0 | fluoroskopie | FALSE | FALSE | FALSE | recRQg0uRLjCQyXhX | HCC | 1 | 79 | rectZrwf13HJwXqgO | 11-07-19 | TRUE | I | 1 | 71 | 2455 | small | TRUE |
| 732 | 1 | Emprint | V | 17 | MWA | 0 | fluoroskopie | FALSE | TRUE | FALSE | recrRpZtNY4yrtpBJ | HCC | 1 | 65 | recfI2h6mkYknrpyD | 27-05-14 | FALSE | None | 1 | 43 | 3611 | small | FALSE |
| 767 | 1 | Acculis | III | 10 | MWA | 0 | fluoroskopie | FALSE | FALSE | FALSE | recSVnCmsWzrBaJbf | CRC | 1 | 73 | recGM0UZ1itdx8J89 | 30-01-13 | FALSE | None | 3 | 175 | 4593 | small | FALSE |
| 768 | 1 | Acculis | VI | 10 | MWA | 0 | fluoroskopie | FALSE | FALSE | TRUE | recSVnCmsWzrBaJbf | CRC | 1 | 73 | recGM0UZ1itdx8J89 | 30-01-13 | FALSE | None | 3 | 175 | 4593 | small | FALSE |
| 769 | 1 | Acculis | V | 15 | MWA | 0 | fluoroskopie | FALSE | FALSE | TRUE | recSVnCmsWzrBaJbf | CRC | 1 | 73 | recGM0UZ1itdx8J89 | 30-01-13 | FALSE | None | 3 | 175 | 4593 | small | FALSE |
| 775 | 0 | Acculis | VII | 15 | MWA | 0 | fluoroskopie | FALSE | FALSE | FALSE | recTgjy63uXODDXMU | HCC | 1 | 69 | recH7WQJCQRAzBXJO | 12-06-13 | TRUE | II | 1 | 45 | 1824 | small | TRUE |
| 778 | 1 | Emprint | VIII | 15 | MWA | 0 | fluoroskopie | TRUE | TRUE | FALSE | recTV7TYJQn17ZNIK | HCC | 1 | 59 | recVJUK6adVopEoit | 06-02-18 | FALSE | None | 2 |  | 1760 | small | TRUE |
| 780 | 1 | Emprint | VI | 11 | MWA | 0 | fluoroskopie | TRUE | FALSE | TRUE | recTV7TYJQn17ZNIK | HCC | 1 | 59 | recVJUK6adVopEoit | 06-02-18 | FALSE | None | 2 |  | 1760 | small | FALSE |
| 825 | 1 | Emprint | VII | 24 | MWA | 0 | fluoroskopie | FALSE | FALSE | FALSE | recuvSAZkqtL1DLdX | CRC | 1 | 64 | recimvSCTMnxXBLaR | 20-02-18 | FALSE | None | 1 |  |  | small | TRUE |
| 838 | 1 | Emprint | IVa | 14 | MWA | 0 | fluoroskopie | FALSE | FALSE | FALSE | recvJFGZdLJzRG4fk | Other | 1 | 65 | recjAiYCM7DlNE4ce | 21-10-16 | FALSE | None | 1 | 50 | 2987 | small | FALSE |
| 839 | 0 | Acculis | IVa | 10 | MWA | 0 | fluoroskopie | FALSE | FALSE | FALSE | recvMCa6fmc3XU2mG | HCC | 1 | 60 | recjDfsJOI6PTS2jA | 18-12-13 | FALSE | None | 2 | 120 | 3302 | small | FALSE |
| 840 | 0 | Acculis | V | 31 | MWA | 0 | fluoroskopie | FALSE | FALSE | FALSE | recvMCa6fmc3XU2mG | HCC | 1 | 60 | recjDfsJOI6PTS2jA | 18-12-13 | FALSE | None | 2 | 120 | 3302 | large | FALSE |
| 851 | 1 | Acculis | VII | 21 | MWA | 0 | fluoroskopie | TRUE | FALSE | TRUE | recW9a3aMQegmUltj | HCC | 1 | 62 | recK0NlNlc82iSlqd | 22-11-16 | FALSE | None | 2 | 55 | 3342 | small | TRUE |
| 856 | 1 | Acculis | VII | 13 | MWA | 0 | fluoroskopie | TRUE | TRUE | FALSE | recW9a3aMQegmUltj | HCC | 1 | 62 | recK0NlNlc82iSlqd | 22-11-16 | FALSE | None | 2 | 55 | 3342 | small | TRUE |
| 864 | 1 | Emprint | IVa | 26 | MWA | 0 | fluoroskopie | FALSE | FALSE | FALSE | recWJ7OeR3RzKBlGT | HCC | 1 | 80 | recKAK6RqpLlGzlDN | 08-03-18 | FALSE | None | 1 | 48 | 2367 | small | FALSE |
| 875 | 1 | Acculis | III | 15 | MWA | 0 | fluoroskopie | TRUE | FALSE | TRUE | recwZAYAcDyqOo2uk | HCC | 1 | 58 | recFdhXq30YlETVYW | 26-09-14 | FALSE | None | 1 | 41 | 2012 | small | FALSE |
| 886 | 1 | Emprint | VI | 15 | MWA | 0 | fluoroskopie | FALSE | FALSE | FALSE | recXdNIx6Q76BMPsy | HCC | 1 | 58 | rec0ma6slIW8fX9et | 15-11-16 | FALSE | None | 1 | 56 | 2102 | small | FALSE |
| 901 | 1 | Emprint | VI | 23 | MWA | 0 | fluoroskopie | TRUE | FALSE | FALSE | recy1zwaNB7k3s4dP | HCC | 0 | 71 | recmScONmX16Zq4aJ | 01-12-15 | TRUE | I | 1 | 64 | 3352 | small | FALSE |
| 935 | 1 | Emprint | II | 6 | MWA | 0 | fluoroskopie | TRUE | TRUE | FALSE | recyRImodwWEnYBSE | CCC | 1 | 62 | recmIlE1MSQqjWBPy | 31-01-18 | FALSE | None | 4 | 107 | 3828 | small | FALSE |
| 936 | 1 | Emprint | III | 7 | MWA | 0 | fluoroskopie | FALSE | FALSE | FALSE | recyRImodwWEnYBSE | CCC | 1 | 62 | recmIlE1MSQqjWBPy | 31-01-18 | FALSE | None | 4 | 107 | 3828 | small | FALSE |
| 937 | 0 | Emprint | III | 11 | MWA | 0 | fluoroskopie | TRUE | FALSE | FALSE | recyRImodwWEnYBSE | CCC | 1 | 62 | recmIlE1MSQqjWBPy | 31-01-18 | FALSE | None | 4 | 107 | 3828 | small | FALSE |
| 938 | 0 | Emprint | III | 11 | MWA | 0 | fluoroskopie | FALSE | TRUE | FALSE | recyRImodwWEnYBSE | CCC | 1 | 62 | recmIlE1MSQqjWBPy | 31-01-18 | FALSE | None | 4 | 107 | 3828 | small | FALSE |
| 948 | 1 | Emprint | V | 13 | MWA | 0 | fluoroskopie | TRUE | FALSE | TRUE | recz5JO4FDAGuV7Ky | CCC | 1 | 74 | recv2H8ocZG2qiGNF | 21-11-17 | FALSE | None | 1 | 32 | 1565 | small | FALSE |
| 952 | 1 | Acculis | VII | 12 | MWA | 0 | fluoroskopie | TRUE | TRUE | FALSE | recZnFk93vmjYq8JN | Other | 1 | 50 | recNeiCMCRg5Uo8GH | 19-11-13 | FALSE | None | 1 | 72 | 3575 | small | TRUE |
| 956 | 0 | Acculis | II | 31 | MWA | 0 | fluoroskopie | TRUE | TRUE | FALSE | recZpYszgHfk82krB | CRC | 0 | 30 | recNgBKcP39640kov | 15-05-14 | FALSE | None | 1 | 90 |  | large | FALSE |
| 961 | 1 | Acculis | III | 13 | MWA | 0 | fluoroskopie | TRUE | FALSE | FALSE | recZVOWHqVokbQj9c | HCC | 1 | 74 | recNMrekZhi67Oj66 | 19-12-13 | FALSE | None | 1 | 66 | 2174 | small | FALSE |
| 962 | 1 | Acculis | VIII | 43 | MWA | 0 | fluoroskopie | FALSE | TRUE | FALSE | reczwrkXxVlmFGy1Q | HCC | 1 | 62 | recnn4CA6hf8BEyYK | 19-06-18 | FALSE | None | 1 | 95 | 3650 | large | TRUE |
| 969 | 0 | Emprint | V | 19 | MWA | 0 | fluoroskopie | FALSE | TRUE | FALSE | recRGVTe9V7ijxmEB | HCC | 1 | 59 | recBBFeB07tZD2aNI | rec7HucsLgaZtWafy | TRUE | II | 1 | 45 | 1824 | small | FALSE |
| 971 | 0 | Acculis | IVa | 27 | MWA | 0 | fluoroskopie | TRUE | FALSE | TRUE | recpew18G5nYcS8Y9 | HCC | 1 | 59 | rec2DuGQCvSiaKeKl | rectL2e1s9rsXBY3e | FALSE | None | 1 | 116 | 5113 | small | FALSE |
| 972 | 0 | Acculis | V | 14 | MWA | 0 | fluoroskopie | TRUE | FALSE | FALSE | recj2oj92iRULEsRW | CRC | 0 | 63 | recVEmhI02zVtqy18 | recVaB8q3uKoEgoa4 | FALSE | None | 1 | 90 |  | small | FALSE |
| 975 | 0 | Acculis | II | 18 | MWA | 0 | fluoroskopie | TRUE | FALSE | FALSE | recT2b3vpI5aJDKfo | HCC | 1 | 67 | recaC3ak9HybUpzDf | recb9XBLXTUlNBkFv | TRUE | II | 1 | 86 | 2815 | small | FALSE |
| 977 | 1 | Acculis | II | 21 | MWA | 0 | fluoroskopie | TRUE | FALSE | TRUE | recYijAoxowbs5lVD | CCC | 1 | 56 | recnpxQK0OxqxBqvN | recaCpXlnidRRqjLW | FALSE | None | 1 | 85 | 2547 | small | FALSE |
| 978 | 0 | Acculis | II | 21 | MWA | 0 | fluoroskopie | FALSE | TRUE | TRUE | recBUFyiGHxwVGBfK | HCC | 1 | 71 | recOvVs1VJKMeE9ge | recryMK3GR3N0fdgQ | FALSE | None | 1 | 85 | 3553 | small | FALSE |
| 979 | 0 | Acculis | II | 21 | MWA | 0 | fluoroskopie | TRUE | TRUE | FALSE | recSsJrogvTbSIVtb | CRC | 1 | 58 | recYgnTmLd7yCkwO7 | recNccSzr1A6WNdy1 | FALSE | None | 1 | 101 | 1685 | small | FALSE |
| 981 | 0 | Emprint | II | 17 | MWA | 0 | fluoroskopie | TRUE | FALSE | FALSE | reccKi0w26C3Mror0 | CRC | 1 | 62 | recY95Y0o3vfewA8E | recj9crEgiT1Xd7po | FALSE | None | 1 | 75 | 2716 | small | FALSE |
